# Integrated Network Pharmacology Analysis and Experimental Validation to Investigate the Mechanism of Zhi-Zi-Hou-Po Decoction in Depression

**DOI:** 10.3389/fphar.2021.711303

**Published:** 2021-10-08

**Authors:** Yongtao Bai, Yingchun Zhang, Shuolei Li, Wenzhou Zhang, Xinhui Wang, Baoxia He, Wenzheng Ju

**Affiliations:** ^1^ Department of Pharmacy, Affiliated Cancer Hospital of Zhengzhou University, Henan Cancer Hospital, Zhengzhou, China; ^2^ Phase I Clinical Research Center, Affiliated Cancer Hospital of Zhengzhou University, Henan Cancer Hospital, Zhengzhou, China; ^3^ College of Pharmaceutical Sciences and Chinese Medicine, Southwest University, Chongqing, China; ^4^ Department of Clinical Pharmacology, Affiliated Hospital of Nanjing University of Chinese Medicine, Nanjing, China

**Keywords:** Zhi-Zi-Hou-Po decoction, UFLC-Q-TOF/MS, network pharmacology, depression, CAMP signaling pathway

## Abstract

Zhi-Zi-Hou-Po Decoction (ZZHPD) is a well-known traditional Chinese medicine (TCM) that has been widely used in depression. However, the antidepressant mechanism of ZZHPD has not yet been fully elucidated. The purpose of this study was to explore the pharmacological mechanisms of ZZHPD acting on depression by combining ultra flow liquid chromatography with quadrupole time-of-flight mass spectrometry (UFLC-Q-TOF/MS) and network pharmacology strategy. The chemical components of ZZHPD were identified using UFLC-Q-TOF/MS, while the potential drug targets and depression-related targets were collected from databases on the basis of the identified compounds of ZZHPD. Protein-protein interaction (PPI) network, gene ontology (GO), and Kyoto encyclopedia of genes and genomes (KEGG) pathway enrichment analyses were used to unravel potential antidepressant mechanisms. The predicted antidepressant targets from the pharmacology-based analysis were further verified *in vivo*. As a result, a total of 31 chemical compounds were identified by UFLC-Q-TOF/MS; 514 promising drug targets were mined by using the Swiss Target Prediction; and 527 depression-related target genes were pinpointed by the GeneCards and OMIM databases. STRING database and Cytoscape’s topological analysis revealed 80 potential targets related to the antidepressant mechanism of ZZHPD. The KEGG pathway analysis revealed that the antidepressant targets of ZZHPD were mainly involved in dopaminergic synapse, serotonin synapse, cAMP, and mTOR signaling pathways. Furthermore, based on the animal model of depression induced by chronic corticosterone, the regulatory effects of ZZHPD on the expression of MAOA, MAOB, DRD2, CREBBP, AKT1, MAPK1, HTR1A, and GRIN2B mRNA levels as well as the cAMP signaling pathway and monoaminergic metabolism were experimentally verified in rats. Our study revealed that ZZHPD is expounded to target various genes and pathways to perform its antidepressant effect.

## Introduction

Depression, a prevalent mental illness with high economic and social costs, poses a major risk for human health (Shafiee et al., 2018; Wang et al., 2020). Major depressive disorder (MDD) shows the characteristics of “three highs,” that is, high prevalence, high recurrence rate, and high disability rate (GBD 2015 Disease and Injury Incidence and Prevalence Collaborators, 2016; Khosravi et al., 2020). From 1990 to 2017, the prevalence of depression in China increased from 3,224.6 per 100,000 to 39,905/100,000, with an increased rate of 24.7%. Among the cases, women and adults over 55 years of age are at a high risk of depression, which is a huge challenge to China’s healthcare (Ren et al., 2020). Although diverse hypotheses have been suggested, including monoaminergic deficiency, immune system disorders, hypothalamic-pituitary-adrenal axis (HPA) unbalance, neurotrophic factors reduction, and neuroplasticity pathway impairment, the exact neurobiological mechanism of depression is still unclear (Dean and Keshavan, 2017; Robson et al., 2017; Levy et al., 2018). In current clinical practice, most antidepressants primarily act by targeting the monoamine neurotransmitter system, such as fluoxetine or venlafaxine. However, these drugs often lead to obvious adverse effects, such as fractures, upper gastrointestinal bleeding, adverse drug reactions, and withdrawal symptoms (Coupland et al., 2018; Hengartner et al., 2019). Thus, it is necessary to find an alternative antidepressant with low toxicity and high efficiency.

As we know, TCM has been used in clinic for thousands of years in China and has played an important role in people’s health. At present, TCM is still an efficient and reliable resource for drug discovery (Cheung, 2011; Han et al., 2020). Many studies have shown that TCM has broad prospects in the prevention and treatment of major depression disorder (Sun et al., 2018; Ye et al., 2019). Zhi-Zi-Hou-Po Decoction (ZZHPD), being composed of the stem, branch, and root barks of *Magnolia officinalis* Rehder & E.H.Wilson (Hou-Po, HP), the ripe fruit of *Gardenia jasminoides* J.Ellis (Zhi-Zi, ZZ), and the young fruit of *Citrus × aurantium* L. (Zhi-Shi, ZS), is a classic prescription for relieving restlessness and removing fullness in the TCM masterpiece “Treatise on Febrile and Miscellaneous Diseases,” compiled by Zhang Zhongjing (AD 150–219) (Wang and Feng, 2019; Zhang and Feng, 2020). ZZHPD has often been utilized in clinical practice to treat mental disorders, especially major depression disorder (Luo and Feng, 2016). There is accumulating evidence that ZZHPD can significantly improve depressive symptoms by balancing energy metabolism, monoaminergic system, and amino acid metabolism and enhancing hippocampal neurogenesis (Xing et al., 2015; Xing et al., 2019). In addition, some studies have found that a single herb of ZZHPD had an antidepressant effect in animals. Two standardized fractions of Zhi-Zi had an antidepressant-like effect associated with BDNF signaling in mice (Ren et al., 2016; Ruan et al., 2019). Acute treatments with Hou-Po extract could attenuate the forced swim-induced experimental depression in mice (Nakazawa et al., 2003), and Zhi-Shi aqueous extract was proved to have an antidepressant effect *in vivo* and *in vitro* (Wu M. et al., 2015). Although the effects of ZZHPD and the single herb on depression have been studied, the antidepressant mechanism has not yet been systematically clarified because of the complexity of ZZHPD with multiple compositions.

Network pharmacology is a comprehensive approach for modern TCM research that integrates multiple disciplines including bioinformatics, systems biology, and traditional pharmacology (Zhang R. et al., 2019). As a new methodology, network pharmacology conforms to the holistic view and the principle of syndrome differentiation and treatment system of TCM, thus providing different research thoughts and feasible technical approaches for probing into the underlying mechanism of Chinese herbal formulae (Banerjee et al., 2019; Yu et al., 2019).

**FIGURE 1:**
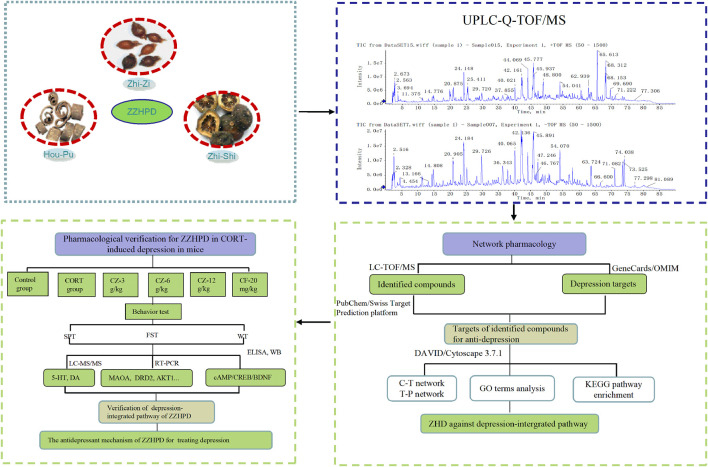
Workflow for dissecting the antidepressant mechanisms of ZZHPD on depression.

In this context, firstly, we used the UFLC-Q-TOF/MS to identify the chemical composition of alcohol extracts of ZZHPD. Next, we mined the potential mechanisms in the treatment of depression using network pharmacology based on the identified phytochemical components of ZZHPD. Several databases were used to collect information in order to predict the phytochemical compound targets, depression-related molecular targets, molecular signaling, and network construction. Finally, a rat depression model induced by corticosterone injection was established to verify the predicted antidepressant targets from pharmacology-based analysis (Figure 1). This study showed that the antidepressant activity of ZZHPD involves the regulation of the expression of MAOA, MAOB, DRD2, CREBBP, AKT1, MAPK1, and HTR1A mRNA levels, as well as the monoaminergic system and cAMP signaling pathway. The present study was the first to use UFLC-Q-TOF/MS-based network pharmacology to explore the possible antidepressant mechanisms of ZZHPD, thereby improving the understanding of this representative antidepressant herbal prescription, which will help to extend its further practical application for patients with depression.

## Materials and Methods

### Materials and Reagents

Reference substance synephrine, geniposide, naringin, neohesperidin, geniposidic acid, chlorogenic acid, caffeic acid, hesperidin, rutin, honokiol, magnolol, and deacetyl asperulosidic acid methyl ester (purity > 98% by HPLC, respectively) were obtained from Chengdu Munster Biotechnology Co., Ltd. (Chengdu, Sichuan, China). Genipin 1-gentiobioside (99.1% by HPLC) was supplied by Shanghai Yuanye Bio-Technology (Shanghai, China). Formic acid (MS-grade), dopamine (DA), and serotonin (5-HT) were from Sigma-Aldrich (St. Louis, MO, United States). The ripe fruit of *Gardenia jasminoides* J. Ellis was originally harvested in Hubei and the stem bark of *Magnolia officinalis* Rehder and E. H. Wilson and the young fruit of *Citrus × aurantium* L. were grown in Sichuan, and they were provided by Beijing Tongrentang Pharmacy (Beijing, China) with batch numbers 20150301, 20150301, and 150401, respectively, and were authenticated by Dr. Qian Zhang from the Jiangsu Province Hospital of Chinese Medicine (Nanjing, Jiangsu, China). Fluoxetine (Flu) was obtained from Lilly Co., Ltd. (Suzhou, Jiangsu, China). Corticosterone was supplied by Tokyo Chemical Industry Co., Ltd. (Shanghai, China). Methanol, acetonitrile, and pyridine (MS-grade) were supplied by Merck Company (Darmstadt, Germany). The ultrapure water was produced by a Milli-Q purification instrument (Milford, MA, United States). And other reagents used in the experiment were of analytical grade and purchased from commercial reagent companies.

### Standard Sample Preparation

Reference standards were precisely weighed using a Sartorius balance with 1/100,000 accuracy and diluted in methanol to prepare stock solutions except for hesperidin, which was dissolved in methanol-pyridine solution (50:50). Before use, these individual reference stock solutions were mixed according to an appropriate amount of each standard sample and then diluted in methanol to obtain reference standard solution.

### Sample Preparation

ZZHPD samples were prepared according to a previously developed method in our laboratory (Bai et al., 2019). Raw medicinal materials were crushed into pieces and sieved through a 40 mesh sieve before extraction. The ethanol extract of ZZHPD was prepared by reflux extraction method with 75% ethanol two times (1 h each) with the ratio 1:1.2:1 of Zhi-Zi, Hou-Po, and Zhi-Shi. The two filtrates were mixed and condensed to 1.2 g of raw materials per ml alcohol extract. Then, an accurate amount of 1 ml of the alcohol extract and 79 ml of acetonitrile water (50:50) was mixed and sonicated (250 W, 50 kHz) for 30 min. Afterward, the herbal samples were diluted with acetonitrile water (50:50) to 100 ml volume, aliquoted into 1.5 ml test tubes, and centrifuged twice at high speed of 12,000 rpm for 10 min at 4°C. The supernatant was collected and passed through 0.22 μm filter membrane and injected with 5 μl for UFLC-Q-TOF/MS analysis.

### LC-TOF/MS Analysis

LC-MS analysis of ZZHPD was implemented on a Shimadzu UFLC 20ADXR instrument matched with a SIL-20AD-XR automatic sampler, CTO-20AC column oven, and 20AD-XR binary pump, and liquid chromatographic analysis was performed using an Agilent ZORBAX SB-C_18_ column (5 μm, 250 mm × 4.6 mm i.d.). The mobile phase, consisting of acetonitrile (component A) and 0.1% formic acid water solution (component B), was run at the rate of 1 ml/min. A gradient elution process is as follows: 2–12% A (0–20 min), 12–20% A (20–40 min), 20–28% A (40–50 min), 28–62% A (50–70 min), and 62–64% A (70–80 min). The column temperature was maintained at 40°C throughout the separation process. In addition, an AB SCIEX Triple TOF™ 5600 equipment (Foster City, CA, United States) coupled with a mass spectrometer with electrospray ion source (ESI) was used for qualitative analysis for ZZHPD. The mass spectrometer was operated in both positive and negative ion modes with dynamic background subtraction, high sensitivity modes, and trigger information-dependent acquisition. The parameters of scan mode were set as follows: auxiliary gas 60 psi, nebulizer gas 60 psi, curtain gas 35 psi, ion source temperature 600°C, and spray voltage 4.5 kV in the positive mode and 4 kV in the negative mode. The ESI mass spectra were acquired in full-scan mode from 50 to 1,500 Da. The Peak View™ Software V.2.0 (Foster City, CA, United States) was used for all the data acquisition and analysis.

### Determination of ZZHPD

The quantitative analysis was performed on an Agilent 1100 system equipped with quaternary pump, autosampler injector, and diode array detector. The chemical compositions were separated on a ZORBAX SB-C_18_ chromatographic column (250 mm × 4.6 mm, 5 μm). The mobile phase was composed of acetonitrile and 0.1% formic acid water as above gradient elution. The flow rate and chromatographic column temperature were set at 1.0 ml/min and 40°C, respectively. The detection wavelength was set at 254 nm and the injection volume was set at 10 μl.

### Identification of Chemical Composition

A database containing the information on the chemical composition of raw materials of ZZHPD was established. The database included the compound names, molecular weight, compound formulae, and compound structures retrieving from the databases including TCMSP (https://tcmspw.com/tcmsp.php) (Guo et al., 2020), PubMed (https://www.ncbi.nlm.nih.gov/pubmed), Web of Science (http://apps.webofknowledge.com), and PubChem (https://www.ncbi.nlm.nih.gov/pccompound) databases. The reference solutions were used to obtain MS/MS fragment ions and retention time under the above-mentioned mass spectrometry conditions, and the unknown compounds were analyzed and identified using the XIC Manager function build in the PeakView software by referring to the established database and literature retrieval.

### Prediction of Potential Targets and Screening of Depression-Related Targets

PubChem database (https://pubchem.ncbi.nlm.nih.gov/) was been utilized to collect the canonical SMILES of these components and was used to foreshadow their molecular targets on the Swiss Target Prediction platform (www.swisstargetprediction.ch/) (Gao et al., 2020). Putative targets were further verified by using the UniProt database (https://www.uniprot.org/). Depression-related targets were obtained by using the keyword “Depression” in the GeneCards (www.genecards.org/) and OMIM databases (www.omim.org/) (Jiang et al., 2020).

### Construction of Component-Target Network and Protein-Protein Interaction (PPI)

Component-target network was established by inputting these potential targets of 31 identified components into the Cytoscape software (version 3.7.1) (Xie et al., 2021). PPIs were discerned utilizing the STRING database (https://string-db.org/) (Zhai et al., 2019). Potential target proteins for the antidepressant components of ZZHPD were imported into this STRING database by selecting “Homo sapiens” proteins only. The final result was saved in *TSV* format and input into the Cytoscape software for the construction of PPI mapping.

### Pathway Analysis

The Database for Annotation, Visualization, and Integrated Discovery (DAVID) (Shen et al., 2020) (https://david.ncifcrf.gov/summary.jsp) was employed for gene ontology (GO) and Kyoto encyclopedia of genes and genomes (KEGG) pathway enrichment analyses of the corresponding targets of each of the identified ZZHPD components. The threshold of *p* < 0.05 was set, the top 20 of biological processes or pathways were selected and imported into the RStudio software for plotting data, and the top 20 pathways were selected to construct the target-pathway network and associated target proteins to import into the Cytoscape software. The KEGG Mapper software (https://www.kegg.jp/kegg/tool/map_pathway1.html) was used to obtain the antidepressant pathway mapping of the ZZHPD.

### Animals and Treatment

Healthy Sprague-Dawley rats (150∼200 g, certificate number SCXK–(SU) 2014–000) were provided by the Animal Experimental Center of Nantong University (Nantong University). Experimental rats were fed on a 12 h light/dark cyclic lighting schedule in controllable temperature and humidity conditions, with *ad libitum* access to food and water. After 7 days of acclimatization, the rats were randomly assigned into six groups (8 rats per group), i.e., control group, corticosterone group (CORT group), corticosterone plus 3 g/kg ZZHPD (CZ-3 g/kg), corticosterone plus 6 g/kg ZZHPD (CZ-6 g/kg), corticosterone plus 12 g/kg ZZHPD (CZ-12 g/kg), and corticosterone plus 20 mg/kg fluoxetine group (CF-20 mg/kg). As previously described, CORT was administered subcutaneously to induce depression at 20 mg/kg once daily for 21 days (Bai et al., 2018). ZZHPD and fluoxetine were orally administrated each day 30 min prior to the corticosterone injection.

### Body Weight Test

Rat body weight was recorded on days 7, 14, and 21 after the initiation of ZZHPD treatment. The rats were weighed by an electronic scale and the average values of the two measurements were recorded.

### Sucrose Preference Test

An SPT was performed to evaluate anhedonia based on the animal’s natural preference for sweets (Liu et al., 2018). All rats in each group were trained to adapt to 1% sucrose solution in two bottles on the 18th day of the experiment and replace a bottle of sucrose solution with fresh water on the 19th day. After the adaptability, water and food were deprived for 12 h, the SPT lasted 4 h (14:00–18:00), and the positions of two bottles were exchanged at 16:00. Sucrose preference was calculated using the formula as follows:

Sucrose preference (%) = Sucrose consumption (ml)/[Water (ml) and Sucrose consumption (ml)] × 100% (Ali et al., 2015).

### Forced Swim Test

An FST was done as the previous description but with slight modification (Bahi et al., 2014). In brief, on day 21 of the experiment, rats were put into a cylindrical red plastic bucket filled with 30 cm high room temperature water. The test lasted 6 min and during the last 4 min, and each rat was forced to swim without touching the bottom of the bucket. The water was changed with fresh water after every test to reduce the interference from previous tests.

### Hippocampal Samples Collection and cAMP Level

After forced swim test, the rats were euthanized under anesthesia with chloral hydrate. After draining the blood from the abdominal aorta, the whole brain tissue was removed, and the blood on the brain surface was washed with a precooled phosphate-buffered saline solution. Then, these bilateral hippocampi were isolated on an ice bag and stored at −80°C. The concentrations of cAMP in the hippocampal samples were detected using a commercial ELISA kit according to the manufacturer’s instructions (Nanjing Jiancheng Bioengineering Institute, Nanjing, Jiangsu, China).

### LC-MS/MS for 5-HT and Dopamine Levels

The levels of 5-HT and DA in the hippocampal samples were analyzed using an AB Sciex QTRAP 5500 coupled to a Prominence™ UFLC system under a multiple reaction monitoring (MRM) mode. Chromatography was selected on a Waters XBridge BEH amide column (3.5 μm, 2.1 mm × 100 mm i.d.) according to the preexperiment results. The mobile phase consisted of pump A (0.2% formic acid acetonitrile) and pump B (0.2% formic acid solution) with a flow rate of 0.8 ml/min. The gradient elution program was as follows: 0–4 min, 95–50% A; 4–8 min, 50–95% A; 8–9 min, 95–95% A. The injection volumes were 1 μl for 5-HT and DA. The optimal operation conditions were as follows: source temperature (TEM), 550°C; spray voltage, 5,000 V; curtain gas (CUR), 35 psi; ion source gas 1 (GS 1), 55 psi; heater gas (GS 2), 55 psi. The Q1, Q3, declustering potential (DP), dwell time (DT), collision cell exit potential (CXP), and collision energy (CE) values of 5-HT, DA, and isoproterenol (internal standard, IS) were optimally selected by Applied Biosystems/MDS Sciex Analyst software (version 1.5.2). The MRM transitions at 177.1 → 160.1, 154.1 → 137.1, and 212.2 → 194.1 were selected to analyze 5-HT, DA, and IS, and the suitable multiple reaction monitoring values are listed in Table 1.

**TABLE 1:** MRM parameters of 5-HT, DA and internal standards (IS).

Analyte	Precursor ion (m/z)	Product ion (m/z)	Dwell time (ms)	DP (V)	CE (ev)	CXP (V)
5-HT	177.1	160.1	50	130	17	16
DA	154.1	137.1	50	52	17	14
IS	212.2	194.1	50	130	14	14

Hippocampal tissues were accurately weighed and homogenized in PBS solution. These samples were centrifuged at high speed of 12,000 rpm after vortexing for 3 min. 50 μl supernatant was placed in an EP tube, and then 150 μl of 1% formic acid in acetonitrile containing 800 ng/ml isoproterenol was also added and vortex-mixed thoroughly for 3 min. After another high-speed centrifugation, 100 μl aliquot of the solution was transferred to an autosampler vial and analyzed using LC-MS/MS system.

### Quantitative RT-qPCR Analysis

The total mRNA was extracted from the hippocampal tissues using TRIzol following the instructions provided by the manufacturer. RNA purity between 1.8 and 2.1 was considered acceptable as indicated by the ratio of absorbance at 260 and 280 nm using a UV spectrophotometer. RNA was reverse-transcribed to cDNA carried out using the PrimeScript RT reagent kit (TaKaRa) with gDNA Eraser. The reaction was performed on an ABI StepOnePlus Real-Time PCR System. The PCR programs are as follows 95°C for the 30 s, 95°C for 5 s, and 60°C for 60 s. Forty cycles of PCR were performed, and relative gene expression levels were calculated using the 2^−ΔΔCt^ method after normalization to β-actin. The primer sequences are shown in Table 2.

**TABLE 2:** The primers for RT-PCR.

Genes	Forward primer (5-3′)	Reverse primer (5-3′)
MAOA	ACT​GCT​CGG​GAA​TTT​GCG​TA	CAA​ATT​TCC​GTT​CCT​GGC​CG
MAOB	GGG​ACA​GAG​TGA​AGC​TGG​AG	CCC​AAA​GGC​ACA​CGA​GTA​AT
DRD2	CAT​TGT​CTG​GGT​CCT​GTC​CT	GAC​CAG​CAG​AGT​GAC​GAT​GA
CREBBP	ATC​CCA​TAG​ACC​CCA​GTT​CC	CGG​CTG​CTG​ATC​TGT​TGT​TA
AKT1	ACT​CAT​TCC​AGA​CCC​ACG​AC	CCG​GTA​CAC​CAC​GTT​CTT​CT
MAPK1	TCT​CCC​GCA​CAA​AAA​TAA​GG	GCC​AGA​GCC​TGT​TCA​ACT​TC
HTR1A	TGT​TGC​TCA​TGC​TGG​TTC​TC	CCG​ACG​AAG​TTC​CTA​AGC​TG
GRIN2B	GTG​AGA​GCT​CCT​TTG​CCA​AC	TGA​AGC​AAG​CAC​TGG​TCA​TC
β-Actin	AGC​CAT​GTA​CGT​AGC​CAT​CC	CTC​TCA​GCT​GTG​GTG​GTG​AA

### Western Blot Analysis

Total proteins were extracted from the hippocampal tissue using RIPA lysis buffer containing 1× protease and phosphatase inhibitor cocktail. The proteins were loaded on a 12% SDS-PAGE gel and transferred onto 0.45 µm PVDF membrane (Millipore, Billerica, MA, United States). After blocking with 5% nonfat dried milk for 1 h at room temperature, the membranes were incubated with BDNF (1:1,000; Abcam, Cambridge, MA, United States), PKA (1:2,000; CST, Danvers, MA, United States), CREB (1:2,000; CST, Danvers, MA, United States), and p-CREB (1:2,000; CST, Danvers, MA, United States) approximately 12 h at 4°C. Subsequently, the membranes were rinsed thrice with PBS/0.1% Tween-20 (PBST) and then exposed to secondary antibodies 1 h at room temperature. The protein blots were washed with PBST, and immunoreactive signals were visualized by enhanced chemiluminescence equipment. The band densities of the proteins were measured by Image Lab software (Bio-Rad Laboratories, Hercules, CA, United States).

### Statistical Analyses

SPSS 18.0 (IBM, Chicago, IL, United States) was used for testing the differences between multiple groups by ANOVA. A value of *p* < 0.05 was considered statistically significant. Figures were prepared using GraphPad Prism 6.0 software (San Diego, CA, United States).

## Results

### Ingredient Identification of ZZHPD Based on LC-TOF/MS

In order to obtain the high sensitivity and optimized chromatographic conditions of alcohol extracts of ZZHPD, several mobile phase systems including methanol-0.1% formic acid water, acetonitrile-0.1% formic acid water, methanol-water, and acetonitrile water were selected. The results showed that acetonitrile water with 0.1% formic acid had the best separation and abundant signal response regardless of positive and negative ion scanning modes in the optimized gradient mode. Total ion chromatograms of alcohol extracts of ZZHPD in the positive and negative ion modes are represented in Supplementary Figure S1, and the extracted ion chromatographs (EIC) of the qualitative samples of ZZHPD are presented in Supplementary Figures S2–S32. In light of the chromatographic peak retention time, relative molecular mass, typical fragment ions, and structural characteristics, as well as comparison to the reference standards and literature searching, a total of 31 compounds were identified in ZZHPD samples. Of them, 13 compounds were unambiguously confirmed by matching the retention time and fragment ions between the samples and reference chemicals. In addition, the data of ZZHPD compounds including retention time, extraction mass, error values, and MS/MS fragment ion were used to analyze the structure of unknown chemical composition, as shown in Table 3.

**TABLE 3:** Compounds identified in the alcohol extracts of ZZHPD by UFLC-TOF/MS.

Peak no.	RT (min)	Adduct	Extraction mass (Da)	Found mass (Da)	Error (ppm)	MS/MS	Formula	Identification	Origin
1	2.68	+H	168.10191	168.1018	−0.9	150, 135, 107, 91	C_9_H_13_NO_2_	Synephrine*	ZS
2	11.41	−H	391.12459	391.12322	−3.5	229, 211,193, 167, 149	C_16_H_24_O_11_	Shanzhiside (Wu H. et al., 2015)	ZZ
3	11.94	−H	373.11402	373.11209	−5.2	211, 193, 167, 149, 123	C_16_H_22_O_10_	Geniposidic acid*	ZZ
4	13.16	−H	403.12459	403.12221	−5.9	241, 223, 191, 139	C_17_H_24_O_11_	Deacetyl asperulosidic acid methyl ester (Wang et al., 2015)	ZZ
5	14.2	−H	389.10894	389.10669	−5.8	345, 183, 139	C_16_H_22_O_11_	Deacetylasperulosidic acid*	ZZ
6	14.35	−H	403.12459	403.12209	−6.2	371,241, 223,127	C_17_H_24_O_11_	Gardenoside (Wu H. et al., 2015)	ZZ
7	15.41	−H	405.14024	405.13759	−6.5	359, 197, 153	C_17_H_26_O_11_	Shanzhiside methylester (Yue et al., 2013)	ZZ
8	15.73	−H	403.12459	403.12328	−3.2	241, 223,139	C_17_H_24_O_11_	Feretoside (Wang et al., 2015)	ZZ
9	19.17	−H	353.08781	353.08624	−4.4	191	C_16_H_18_O_9_	Chlorogenic acid*	ZZ
10	20.9	−H	549.18249	549.17963	−5.2	225, 207, 123	C_23_H_34_O_15_	Genipin 1-gentiobioside*	ZZ
11	21.04	−H	179.03498	179.03486	−0.7	135	C_9_H_8_O_4_	Caffeic acid*	ZZ
12	24.18	−H	387.12967	387.12733	−6.1	225, 207, 147	C_17_H_24_O_10_	Geniposide*	ZZ
13	24.19	−H	225.07685	225.07675	−0.4	207, 147, 101	C_11_H_14_O_5_	Genipin (Jia et al., 2019)	ZZ
14	34.88	+H	611.16066	611.1601	−1	303	C_27_H_30_O_16_	Rutin*	ZZ
15	40.02	+H	581.18648	581.1853	−2	419, 273, 435	C_27_H_32_O_14_	Narirutin (Ma et al., 2011)	ZS
16	42.16	+H	581.18648	581.1855	−1.6	273, 153	C_27_H_32_O_14_	Naringin*	ZS
17	42.99	−H	577.15628	577.14988	−1.1	269, 577	C_27_H_30_O_14_	Rhoifolin (Li et al., 2013)	ZS
18	44.07	+H	611.19705	611.1954	−2.7	465, 449, 303, 413	C_28_H_34_O_15_	Hesperidin*	ZS
19	45.79	+H	611.19705	611.1954	−2.8	465, 449, 303	C_28_H_34_O_15_	Neohesperidin*	ZS
20	54.04	+H	595.20213	595.2004	−2.9	287, 153	C_28_H_34_O_14_	Poncirin (Ma et al., 2011)	ZS
21	56.84	−H	271.0612	271.05906	−7.9	177, 151, 119	C_15_H_12_O_5_	Naringenin (Li et al., 2013)	ZS
22	60.91	+H	373.12818	373.1274	−2.1	358, 343, 315, 181	C_20_H_20_O_7_	Isosinensetin (Wang et al., 2007)	ZS
23	61.68	+H	217.04954	217.0492	−1.5	202, 174, 146	C_12_H_8_O_4_	Bergapten (Liao et al., 2018)	ZS
24	62.43	+H	473.21699	473.2156	−3.1	369, 187, 161	C_26_H_32_O_8_	Deacetylnomilin (Tian and Schwartz, 2003)	ZS
25	63.2	+H	373.12818	373.1273	−2.3	357, 343, 315	C_20_H_20_O_7_	Sinensetin (Wang et al., 2007)	ZS
26	63.73	+H	471.20134	471.2001	−2.6	425, 161	C_26_H_30_O_8_	Limonin (Tian and Schwartz, 2003)	ZS
27	65.61	+H	403.13874	403.1376	−2.7	388, 373, 358, 330	C_21_H_22_O_8_	Nobiletin (Wang et al., 2007)	ZS
28	66.05	+H	515.22756	515.2255	−4.1	469, 161	C_28_H_34_O_9_	Nomilin (Cai et al., 2016)	ZS
29	68.32	+H	373.12818	373.1271	−2.8	358, 343, 328, 300	C_20_H_20_O_7_	Tangeretin (Wang et al., 2007)	ZS
30	71.07	+H	267.13796	267.137	−3.5	239, 226, 197, 165	C_18_H_18_O_2_	Honokiol*	HP
31	74.03	+H	267.13796	267.1372	−2.7	239, 226, 197, 165	C_18_H_18_O_2_	Magnolol*	HP

* Identifications confirmed with standard compound. Notes: HP, *Magnolia officinalis*; ZZ, *Gardenia jasminoides*; ZS, *Citrus × aurantium*.

### Quantitative Analysis of Seven Compounds

As shown in Supplementary Figure S33, seven compounds in ZZHPD showed a peak shape and reached better baseline separation in 80 min under optimum conditions. They were genipin 1-gentiobioside 2.45 mg/g, geniposide 12.32 mg/g, naringin 21.90 mg/g, hesperidin 1.77 mg/g, neohesperidin 22.17 mg/g, honokiol 1.49 mg/g, and magnolol 4.44 mg/g in ZZHPD.

### Predicted Targets and Component-Target Network

**FIGURE 2:**
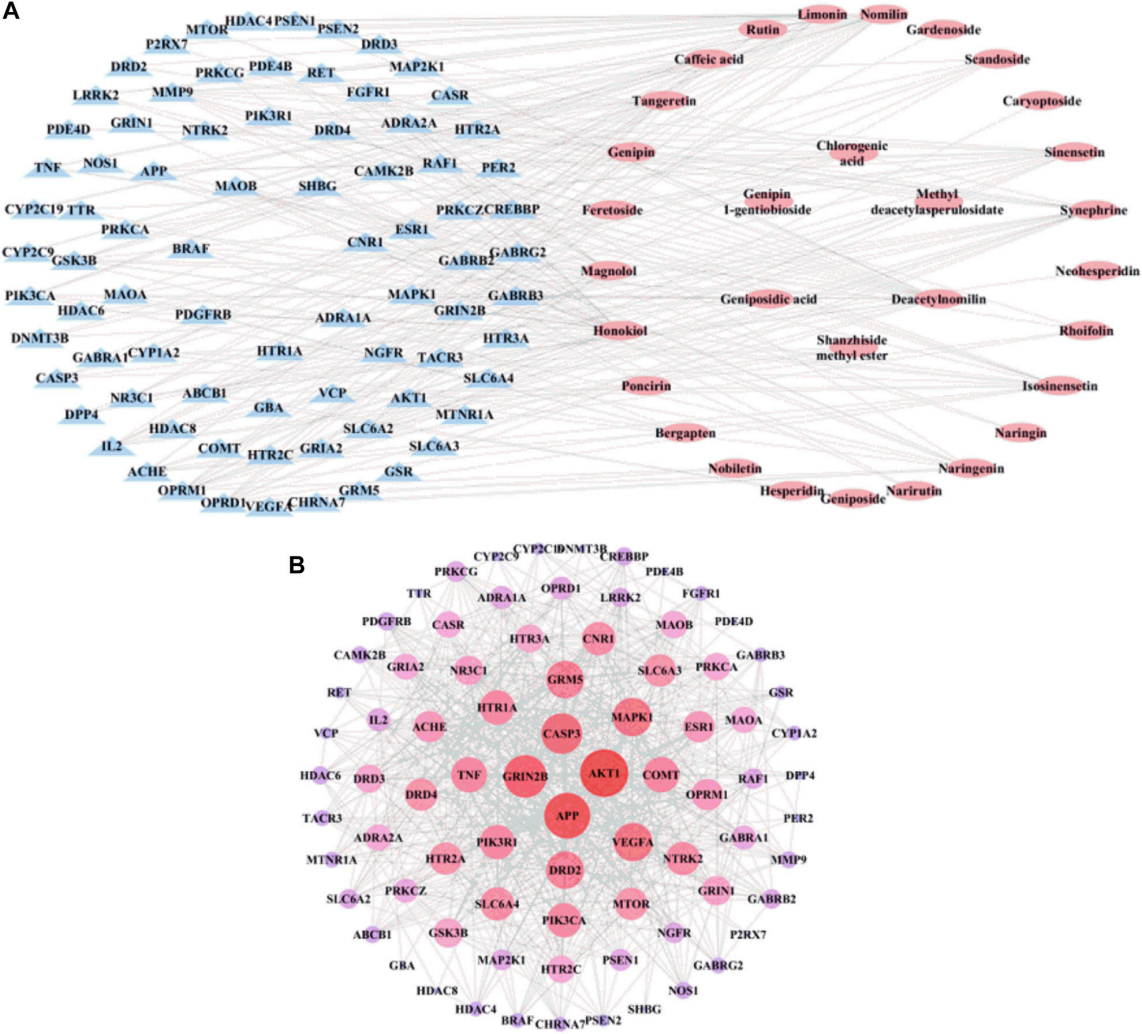
The network construction for ZZHPD components and targets. **(A)** Composition-target network, and **(B)** PPI network related to the active components of ZZHPD.

To understand the pharmacological activities of the identified components in the alcohol extracts, potential molecule targets of these components were predicted using the Swiss Target Prediction platform (Gu et al., 2020; Zhang et al., 2020). A total of 1,484 putative targets closely related to 31 identified components were retrieved, and 514 targets were finally obtained after removing duplicates (Supplementary Table S1). Furthermore, 527 depression-related target genes were pinpointed by the GeneCards and OMIM databases after deleting duplicates. By comparing these depression-related target genes, 80 genes that may be involved in the antidepressant activity of ZZHPD were recorded. Figure 2A shows the component-target network diagram generated by the Cytoscape software. The network involved 31 chemical components, 80 targets, 111 nodes, and 242 edges. The network characteristics included the network density of 0.040, the characteristic path length of 3.295, an average number of adjacent nodes of 4.360, the network heterogeneity of 1.114, and the network centrality of 0.210. The chemical components with a higher degree value were honokiol (degree = 27), deacetylnomilin (degree = 21), nomilin (degree = 21), magnolol (degree = 18), synephrine (degree = 17), and limonin (degree = 17), indicating that these components are important for the therapeutic effect of ZZHPD on depression.

### PPI Network

To further explore the complex mechanism of action of ZZHPD, the PPI relationship was further evaluated by using the Cytoscape software and STRING database to create a PPI network. As shown in Figure 2B **,** the constructed PPI network had 80 targets, 80 nodes, and 700 edges. The size of the node degree value is indicated by the size and color of the nodes; that is, the bigger the node and the closer the color to red, the higher the node degree value. The results showed the network density of 0.222, the characteristic path length of 1.95, an average number of adjacent nodes of 17.5, the network heterogeneity of 0.542, and the network centrality of 0.305. The node degrees of the genes, AKT1, APP, GRIN2B, CASP3, VEGFA, MAPK1, GRM5, and DRD2, were greater than 30, suggesting that these genes as potential targets are involved in the treatment of depression with ZZHPD.

### Gene Function and Pathway Analysis

**FIGURE 3:**
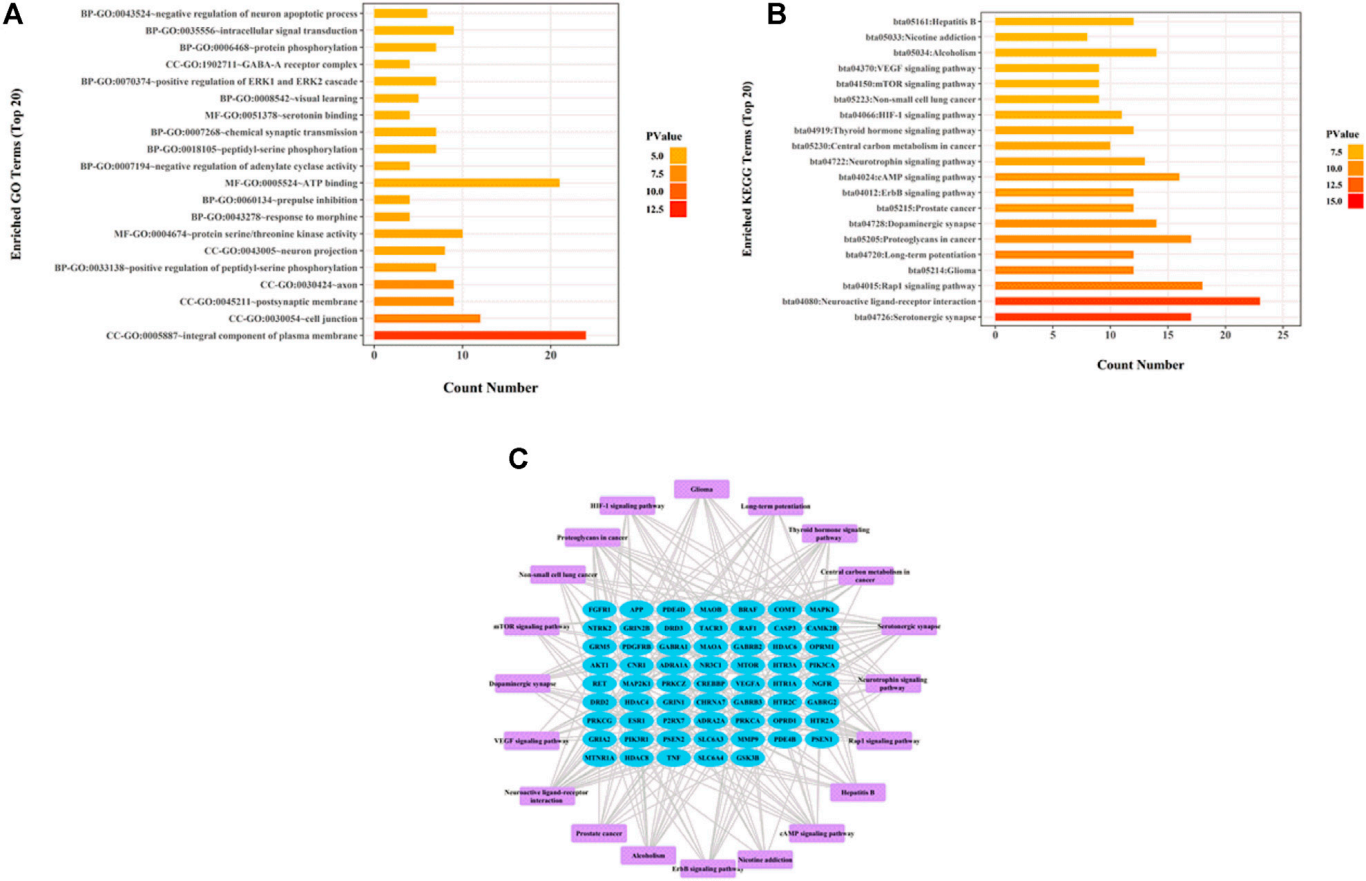
Network pharmacology analysis of thirty-one validated constituents of ZZHPD. **(A)** GO enrichment analysis, **(B)** KEGG enrichment analysis, and **(C)** target-pathway network in the antidepression effects of ZZHPD.

To test whether active components in the alcohol extracts of ZZHPD can modulate target gene-related signaling pathways, DAVID online database was utilized to analyze GO and KEGG pathway enrichment in the biological system networks (Ou et al., 2020). Figures 3A,B showed the enriched GO and KEGG analyses of 80 target genes of 31 active components, respectively (Supplementary Tables S2, S3). The top 20 biological processes or pathways were analyzed and shown. By importing the first 20 KEGG pathways and associated target proteins into the Cytoscape software, the target-pathway network was generated (Figure 3C).

GO enrichment analysis revealed three aspects: cellular components, molecular functions, and biological processes. Cellular components were mostly related to the integral component of plasma membrane, postsynaptic membrane, axon, neuron projection, and GABA-A receptor complex. The biological processes mainly included the positive regulation of peptidyl-serine phosphorylation, prepulse inhibition, negative regulation of adenylate cyclase activity, chemical synaptic transmission, protein phosphorylation, and intracellular signal transduction. Molecular functions were chiefly associated with serine/threonine kinase activity, ATP binding, and serotonin binding protein. KEGG analysis indicated the pathways predominantly involved in the antidepressant effects of ZZHPD, including serotonergic synapse, neuroactive ligand-receptor interaction, dopaminergic synapse, cAMP signaling pathway, and mTOR signaling pathway.

### Effects of ZZHPD on Body Weight, Sucrose Preference Test, and Forced Swim Test

**FIGURE 4:**
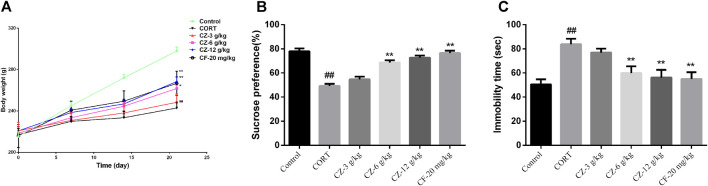
The synergetic antidepressant effects of ZZHPD in rats subjected to CORT administration. **(A)** Body weight, **(B)** sucrose consumption, and **(C)** immobility time in the forced swim test. All data were represented as the mean ± SD. *n* = 8. Compared with model group, *p < 0.05 and **p < 0.01; compared with the control group, ^#^ *p* < 0.05 ^##^ *p* < 0.01.

After subcutaneous injection of CORT for 21 consecutive days, the model group showed obvious depressive behavior. Compared with the control group, the CORT group had a marked reduction in body weight and sucrose intake rate (*Ps* < 0.01) and a significantly longer immobility time during the FST experiment (*p* < 0.01), indicating that the CORT-induced depression model was successfully constructed. The ANOVA test showed that ZZHPD produced significant effects on SPT and FST in rats. As shown in Figure 4, ZZHPD treatment at the doses of 6 and 12 g/kg/d (*Ps* < 0.01) significantly increased body weight and sucrose uptake rate (*Ps* < 0.01). In addition, ZZHPD decreased the immobility time of FST versus the CORT group.

### Effects of ZZHPD on cAMP, 5-HT, and Dopamine Levels of Hippocampus

**FIGURE 5:**
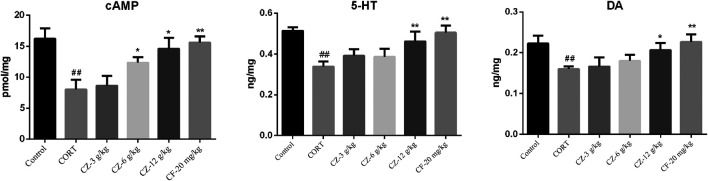
Effects of ZZHPD on cAMP, 5-HT, and DA levels in hippocampi of rats exposed to CORT. All data were represented as the mean ± SD. *n* = 8. Difference was analyzed using one-way ANOVA followed by Dunnett’s post hoc test, *p < 0.05 and **p < 0.01 compared with model group; ^#^ *p* < 0.05 and ^##^ *p* < 0.01 compared with the control group.

Varying cAMP levels in hippocampal tissue were shown in Figure 5. ZZHPD at a low dose (3 g/kg) had no effect on cAMP levels, while medium and high doses of ZZHPD (6 and 12 g/kg; Ps < 0.05) significantly increased the cAMP levels in the hippocampus compared with the model group. As expected, the positive control (fluoxetine) significantly increased the cAMP levels (*p* < 0.01). Hippocampal 5-HT and DA levels in the model group were significantly lower than those in the control group, but they were significantly increased following ZZHPD treatment at a high dose of ZZHPD (*p* < 0.05). In addition, elevated levels of 5-HT and DA were observed with fluoxetine treatment (*Ps* < 0.01).

### RT-qPCR Detection of Related Gene Expression

**FIGURE 6:**
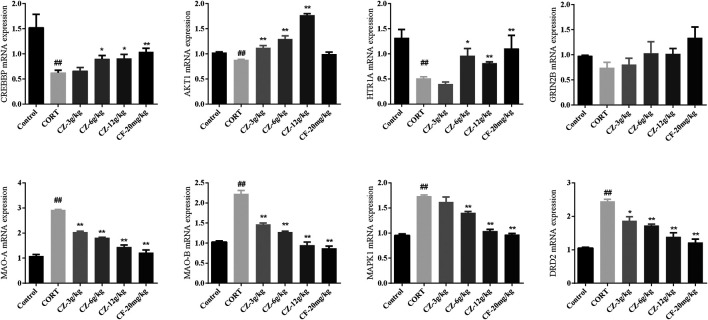
Effects of oral administration of ZZHPD on eight genes detected by RT-qPCR. All data were expressed as the mean ± SD. *n* = 3. *p < 0.05 and **p < 0.01 compared with model group; ^#^ *p* < 0.05 and ^##^ *p* < 0.01 compared with the control group.

The results of RT-qPCR indicated that the expression levels of CREBBP, AKT1, and HTR1A were significantly lower, while those of MAOA, MAOB, DRD2, and MAPK1 were higher in the model group than in the control group. As shown in Figure 6, the group undergoing ZZHPD treatment showed a marked increase in CREBBP, AKT1, and HTR1A mRNA levels. Meanwhile, the mRNA levels expression of MAOA, MAOB, DRD2, and MAPK1 in the ZZHPD-treated group were significantly attenuated compared with those in the CORT group.

### Effects of ZZHPD on Protein Expression of PKA, BDNF, and p-CREB

**FIGURE 7:**
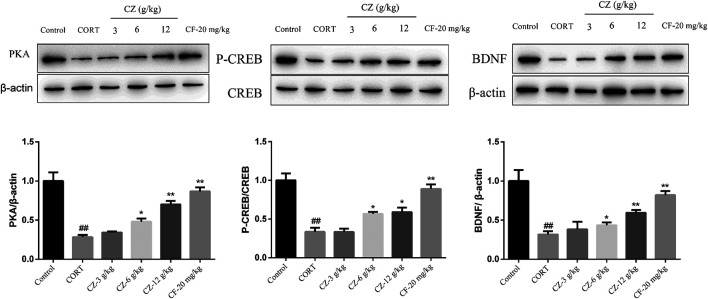
Effects of ZZHPD on PKA, CREB, p-CREB, and BDNF protein expression in the hippocampus of CORT-treated rats. All data were expressed as the mean ± SD. *n* = 3. *p < 0.05 and **p < 0.01 compared with model group; ^#^ *p* < 0.05 and ^##^ *p* < 0.01 compared with the control group.

In order to further explore the antidepressant mechanism of ZZHPD, cAMP signaling pathway was studied by detecting the protein expression levels of PKA and BDNF and the phosphorylation status of CREB by WB. As shown in Figure 7, the model group rats showed marked downregulation of the expression levels of PKA, BDNF, and phosphorylated CREB compared with the control group. Meanwhile, the protein expression levels of PKA, p-CREB, and BDNF increased significantly following treatment with medium and high doses of ZZHPD.

## Discussion

MDD has complex and multifactorial etiology related to psychological, physical, emotional, and social impairment (Leubner and Hinterberger, 2017; Li et al., 2021). At present, effective treatments for depression are very limited, and TCM is gaining more attention for the clinical therapy of depression (Wang et al., 2019). However, the clinical effects of TCM are inconsistent and difficult to evaluate due to variable composition resulting from a nonstandard preparation process in certain settings. Moreover, the efficacy of TCM depends largely on the combination of multiple active compounds, rather than on any single individual ingredient. These issues pose a big challenge for the evaluation of the clinical efficacy of TCM and for understanding of the material basis and molecular mechanisms responsible for its therapeutic efficacy. Fortunately, network pharmacology has evolved from systems biology and multidirectional pharmacology, thus revolutionizing the practice of TCM by shifting the paradigm of “one drug, one target, one disease” towards the model of “multicomponent, multitarget, multidisease,” therefore making it possible to uncover the complex relationship between active components and their targets (Wang J. et al., 2018; Yu et al., 2018; Sun et al., 2021).

ZZHPD is an effective TCM formula used for the treatment of depression. Yao et al. utilized FST and TST to assess the antidepressant effect of ZZHPD after the last administration. The results demonstrated that the antidepressant-like mechanism of ZZHPD was related to the monoaminergic system (Yao et al., 2013). However, the chemical composition and the interactions of herbs in ZZHPD during codecoction are unclear. Liu et al. developed an effectively integrated method based on HPLC-MS coupled with chemometrics to identify the compounds in ZZHPD and to reveal the potential physicochemical changes during decoction (Liu et al., 2016). Our study used the UFLC-Q-TOF/MS to identify the chemical composition of alcohol extracts of ZZHPD and mined the potential mechanism in the treatment of depression using network pharmacology. By network analysis, our study obtained an initial understanding of potential molecular targets, major signaling pathways, important regulatory processes, and associated cellular components in the treatment of depression with ZZHPD by network analysis. More specifically, the analysis of network pharmacology data suggested that the antidepressant effects of ZZHPD were largely mediated by the regulation of the cAMP signaling pathway and 5-HT/DA synaptic transmission. Indeed, the cAMP/PKA/BDNF signaling pathway is one of the most important signaling pathways involved in antidepressant therapy. cAMP could mediate the protein kinase A-cAMP response element-binding (CREB) signaling pathway as an important second messenger (Wang X. L. et al., 2018). CREB, an important transcriptional element necessary for the survival of neurons, is mainly activated at a particular residue, serine 133 (Ser133). And BDNF is the major transcriptional product of CREB phosphorylation (Chen et al., 2019; Yu et al., 2021), which acts on certain neurons of the central nervous system to support the survival of existing neurons and encourage the growth and differentiation of new neurons and synapses (Kim et al., 2017; Bai et al., 2018). Meanwhile, preclinical and clinical trials have indicated that the related proteins of cAMP/PKA/CREB are downregulated in depression and upregulated by antidepressant therapy (Li et al., 2009; Fujita et al., 2017; Lian et al., 2018).

In addition, several key components including magnolol, synephrine, honokiol, and limonin, may largely contribute to the antidepressant efficacy of ZZHPD by network pharmacology analysis. These active compounds share similar pharmacological effects, although each has unique biological activities. For example, magnolol—the main component of *Magnolia officinalis*—has antioxidative, antitumor, and antidepressant effects *via* suppressing neuroinflammation and decreasing the levels of p-ERK, GSK3β, and Smad (Cheng et al., 2018; Chei et al., 2019), synephrine has antitumor and antidepressant properties through inhibition of Galectin-3-AKT/ERK signaling and the stimulation of α1 adrenoceptors (Song et al., 1996; Xu et al., 2018), and honokiol has been proven to protect against inflammation and depression *via* suppressing the activation of TXNIP-NLRP3 inflammasome and NF-κB signaling pathway, reducing the levels of related proinflammatory cytokines (Tang et al., 2018; Xia et al., 2019; Zhang B. et al., 2019), while limonin has been recognized to have a medicinal value with antioxidant and tumor-inhibiting capacities (Breksa and Manners, 2006; Su et al., 2019). Therefore, the above-mentioned literature supports the idea that these natural products are thought to work synergistically to endow ZZHPD with effective antidepressant effects.

In animal behavior experiments, including body weight test, SPT, and FST, ZZHPD showed antidepressant effects after 3 weeks of treatment. Notably, ZZHPD effectively regulated MAOA, MAOB, DRD2, CREBBP, AKT1, MAPK1, and HTR1A mRNA levels and upregulated BDNF expression by the cAMP signal pathway, involving the phosphorylation of transcription factor CREB. Meanwhile, hippocampal 5-HT and DA contents were obviously improved with ZZHPD treatment. These findings were in accordance with the predictive results derived from the network analysis.

## Conclusion

This study systematically evaluated the antidepressant mechanism of ZZHPD by network pharmacology analysis and experimental verification. UFLC-Q-TOF/MS-based analytical tool was used to profile the phytochemicals of ZZHPD, resulting in the identification of a total of 31 active compounds in the alcohol extracts of ZZHPD. The KEGG pathway enrichment indicated that the antidepressant mechanism of ZZHPD was mainly involved in the dopaminergic synapse, serotonin synapse, and cAMP signaling pathway. The subsequent *in vivo* experiments verified that the antidepressant mechanism of ZZHPD was consistent with the predictions of network pharmacology. In conclusion, we demonstrated that the antidepressant mechanism of ZZHPD is based on multicomponent, multitarget, and system regulation.

## Data Availability

The raw data supporting the conclusion of this article will be made available by the authors, without undue reservation, to any qualified researcher.
