# Supplementary material for: Integrated Network Pharmacology Analysis and Experimental Validation to Investigate the Mechanism of Zhi-Zi-Hou-Po Decoction in Depression
Source: Front Pharmacol. 2021 Oct 8;12:711303. doi: 10.3389/fphar.2021.711303 (PMC8531485; doi:10.3389/fphar.2021.711303)
Supplement: Supplementary file 1 [file DataSheet2.docx]

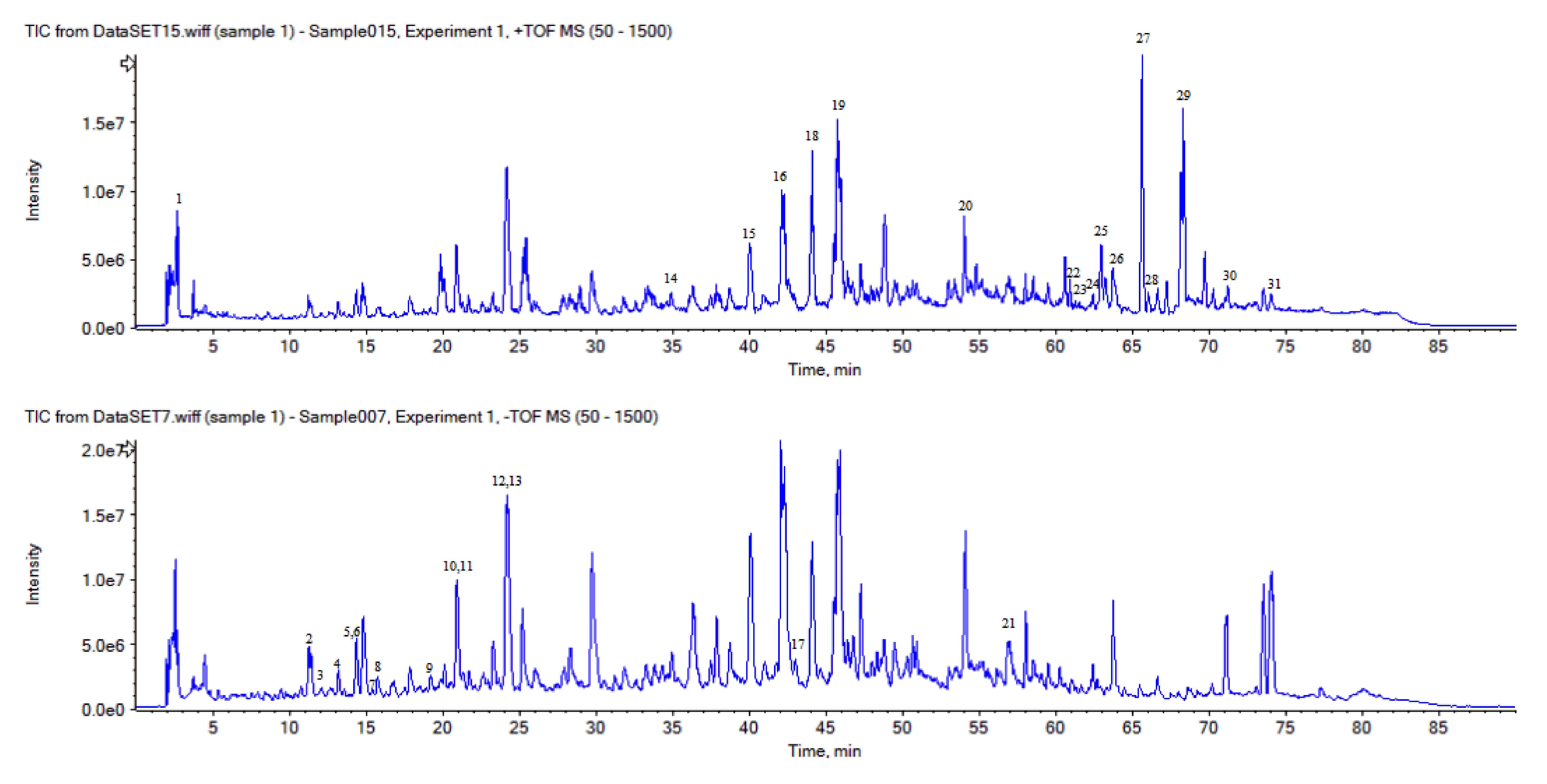


Fig. S1. The total ion chromatograms of ZZHPD acquired by UPLC-TOF/MS with thirty-one validated constituents labeled in positive mode and negative mode.


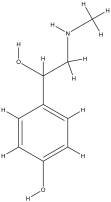

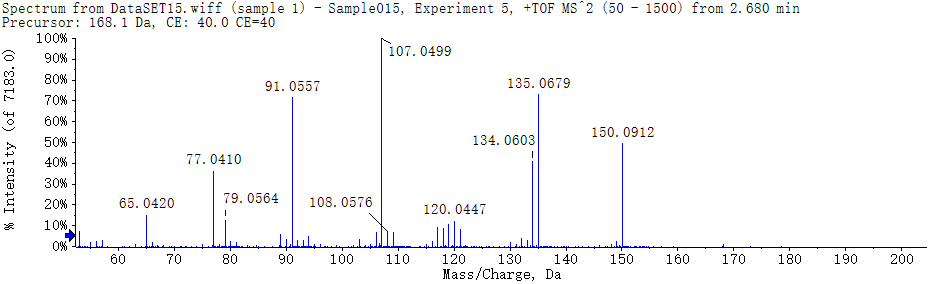


Fig. S2. Secondary mass spectrogram of component synephrine


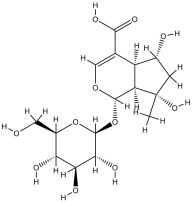

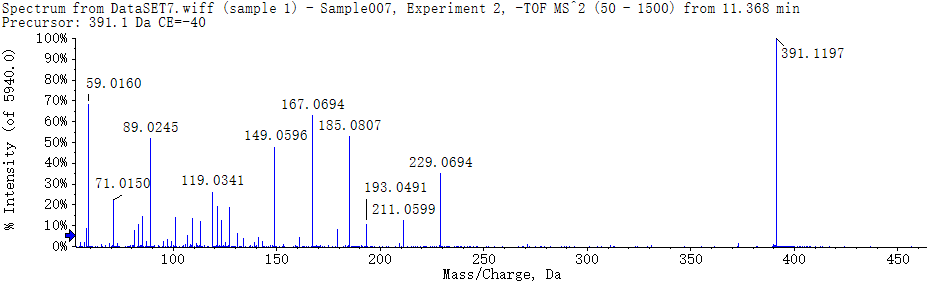


Fig. S3. Secondary mass spectrogram of component shanzhiside


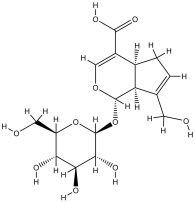

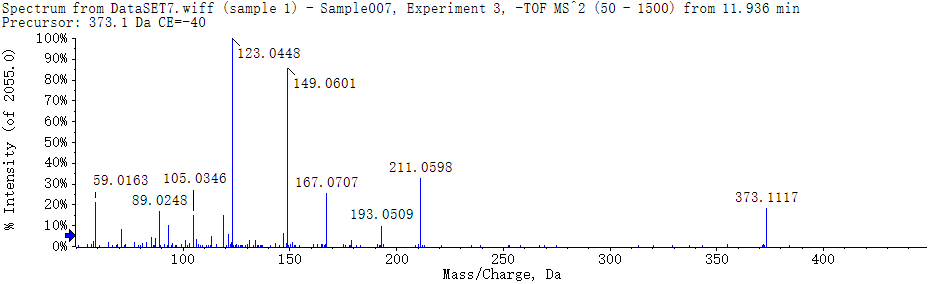


Fig. S4. Secondary mass spectrogram of component geniposidic acid


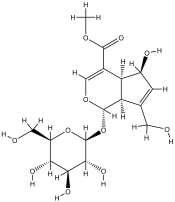

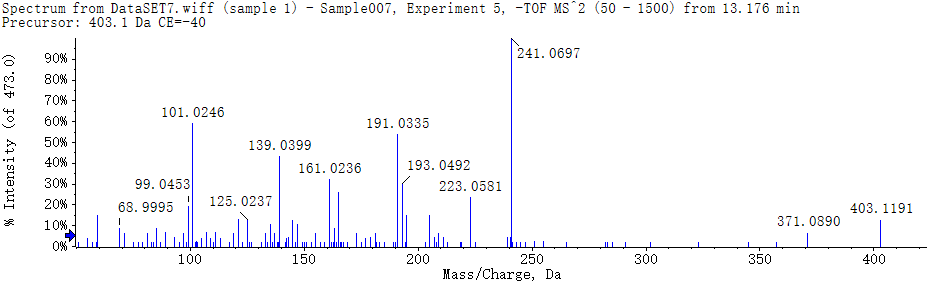


Fig. S5. Secondary mass spectrogram of component deacetyl asperulosidic acid methyl ester


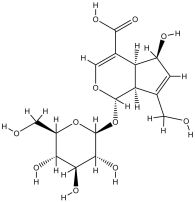

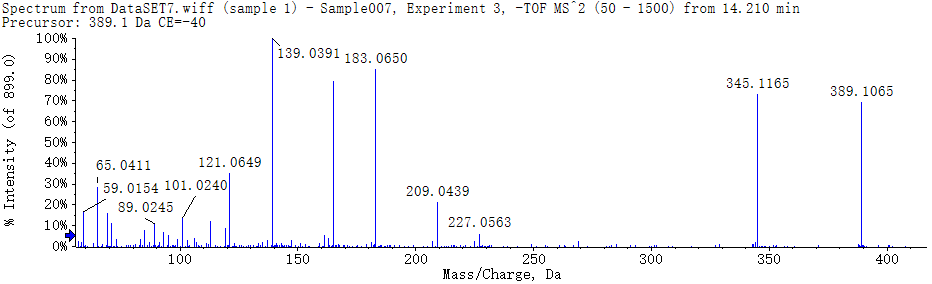


Fig. S6. Secondary mass spectrogram of component deacetylasperulosidic acid


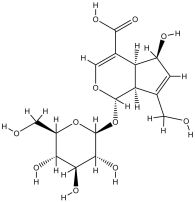

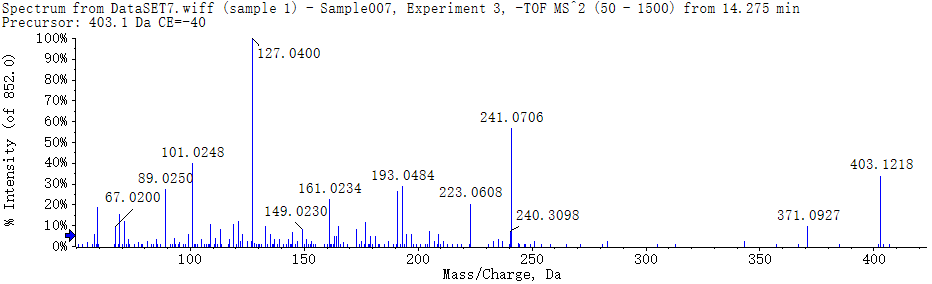


Fig. S7. Secondary mass spectrogram of component gardenoside


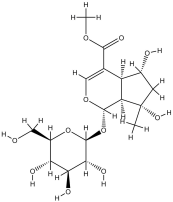


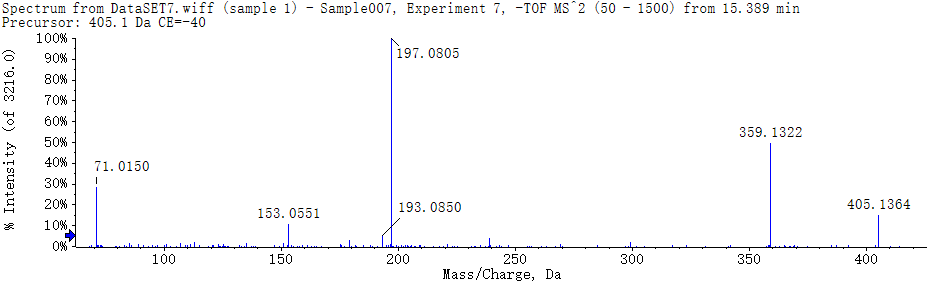


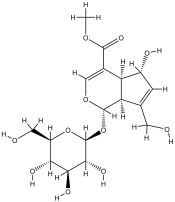
Fig. S8. Secondary mass spectrogram of component shanzhiside methylester


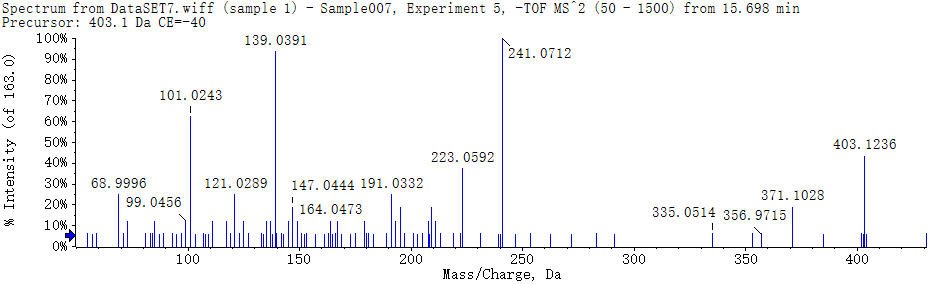


Fig. S9. Secondary mass spectrogram of component feretoside


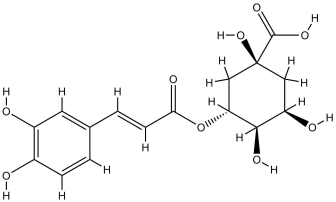

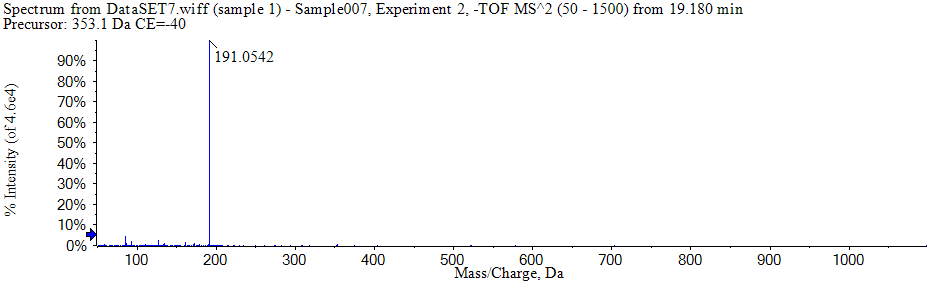


Fig. S10. Secondary mass spectrogram of component chlorogenic acid


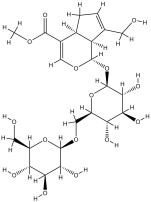


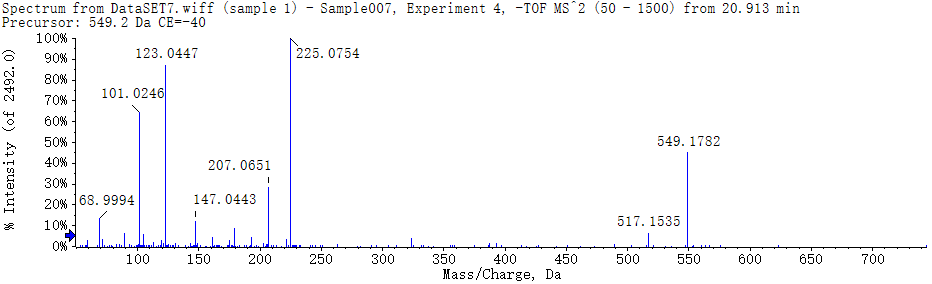


Fig. S11. Secondary mass spectrogram of component genipin 1-gentiobioside


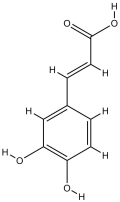


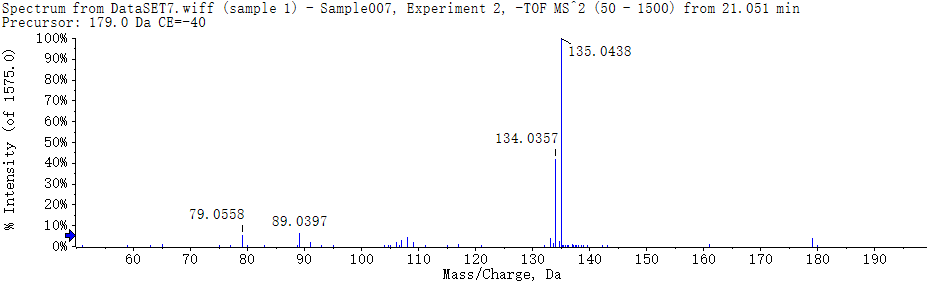


Fig. S12. Secondary mass spectrogram of component caffeic acid


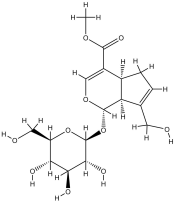


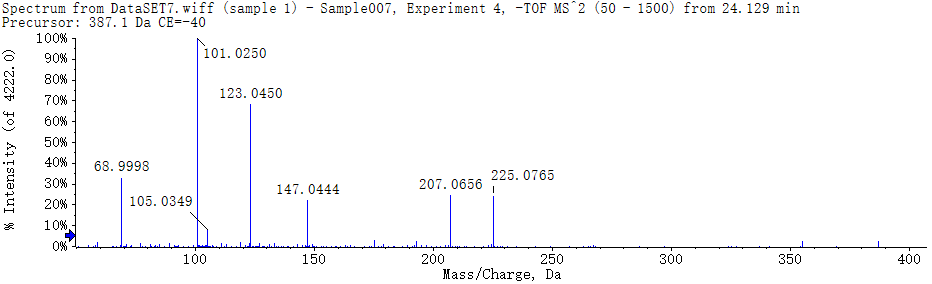


Fig. S13. Secondary mass spectrogram of component geniposide


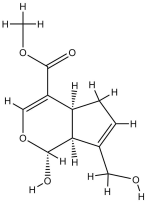

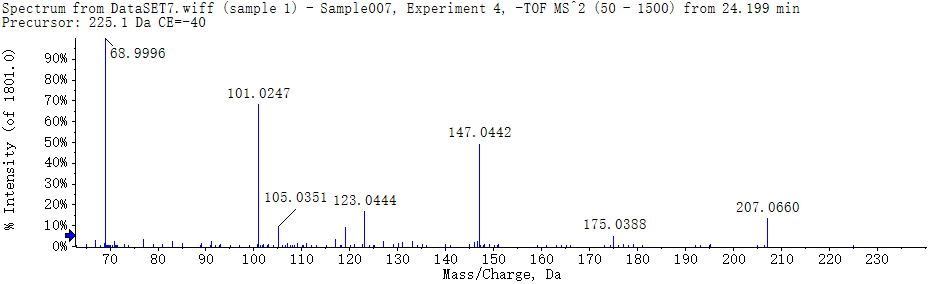


Fig. S14. Secondary mass spectrogram of component genipin
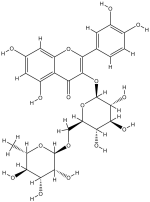


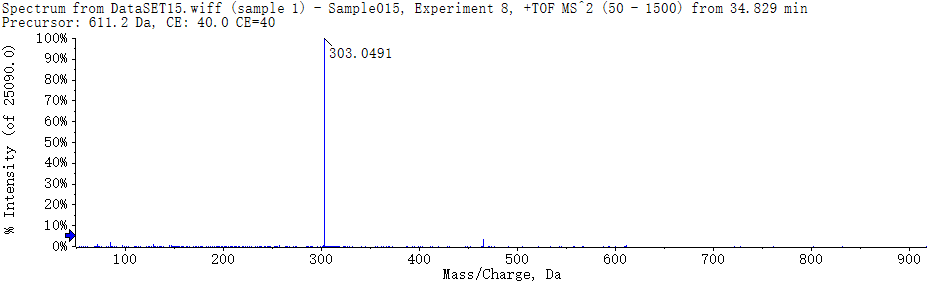


Fig. S15. Secondary mass spectrogram of component rutin


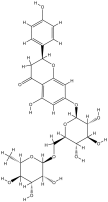


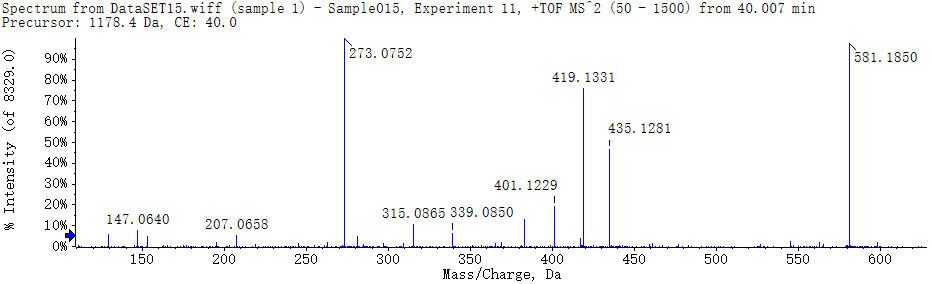


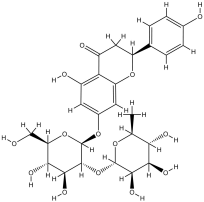
Fig. S16. Secondary mass spectrogram of component narirutin


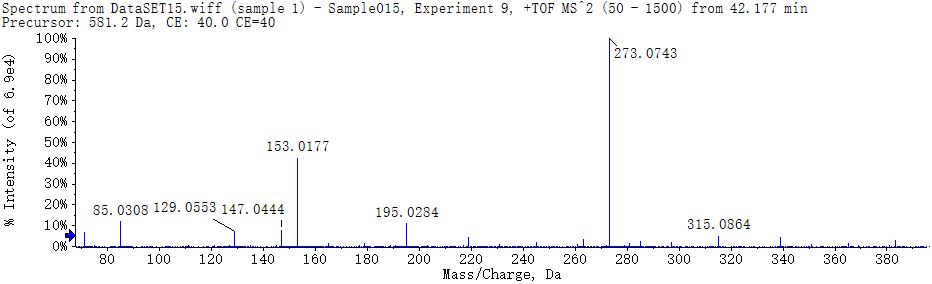


Fig. S17. Secondary mass spectrogram of component naringin


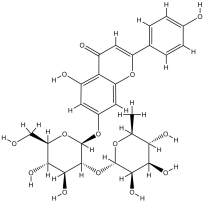

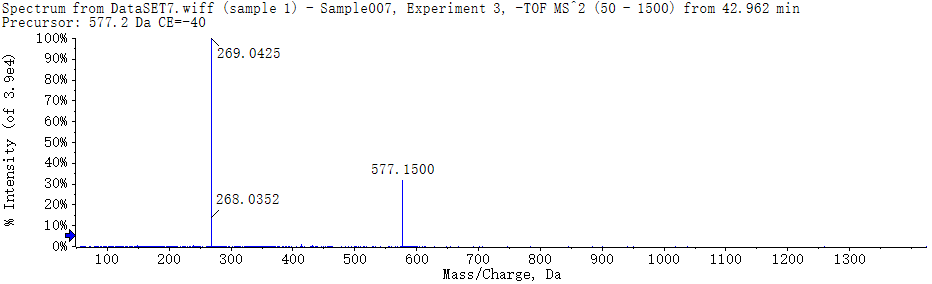


Fig. S18. Secondary mass spectrogram of component rhoifolin


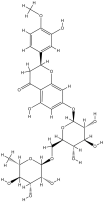


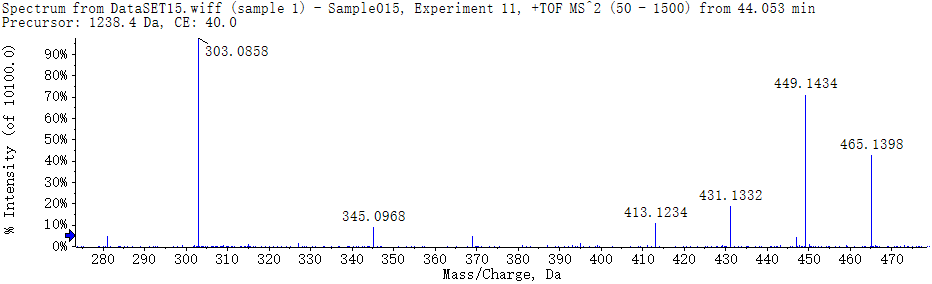


Fig. S19. Secondary mass spectrogram of component hesperidin


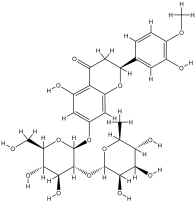

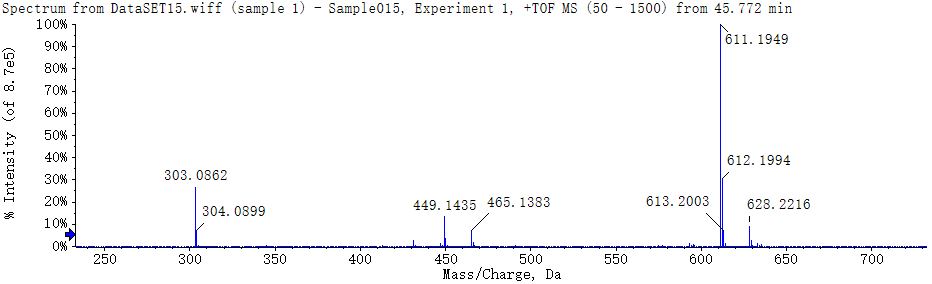


Fig. S20. Secondary mass spectrogram of component neohesperidin


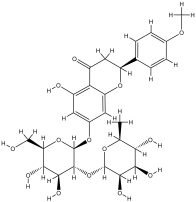


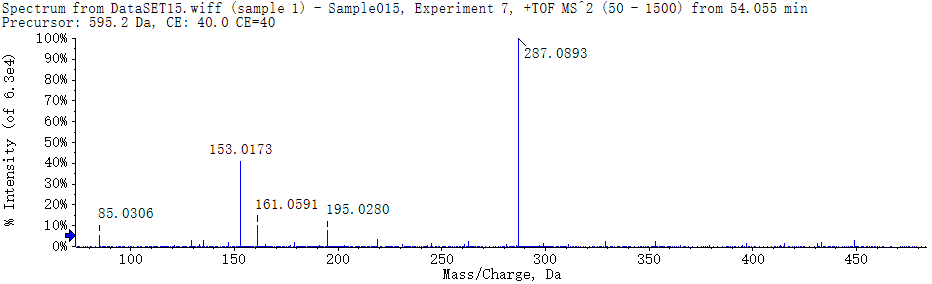


Fig. S21. Secondary mass spectrogram of component poncirin


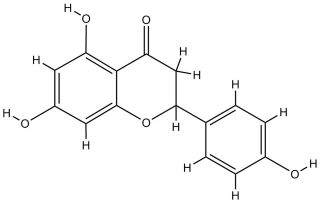

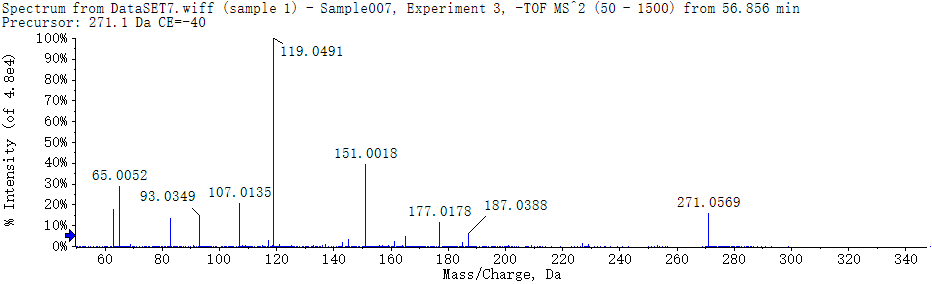


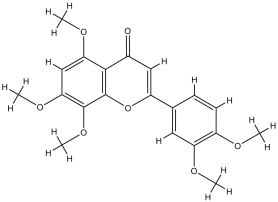
Fig. S22. Secondary mass spectrogram of component naringenin


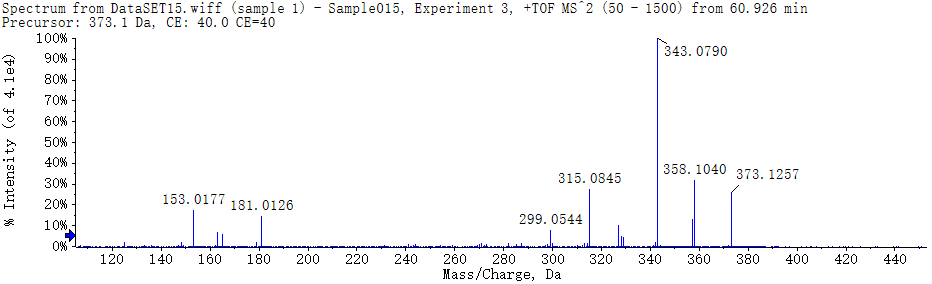


Fig. S23. Secondary mass spectrogram of component isosinensetin


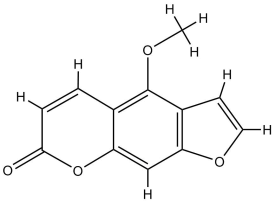

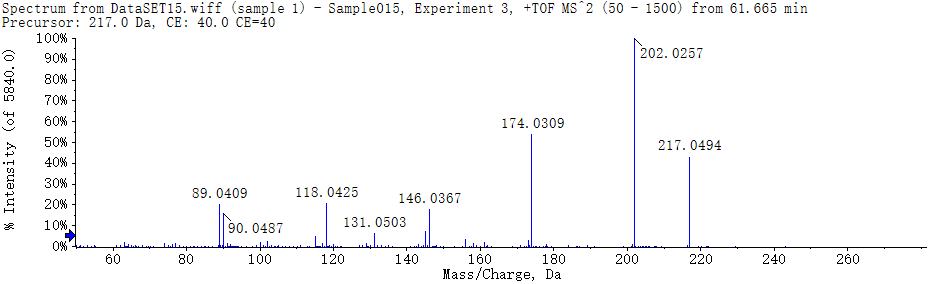


Fig. S24. Secondary mass spectrogram of component bergapten


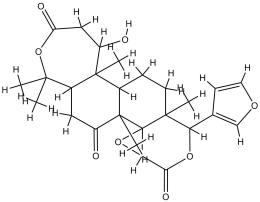

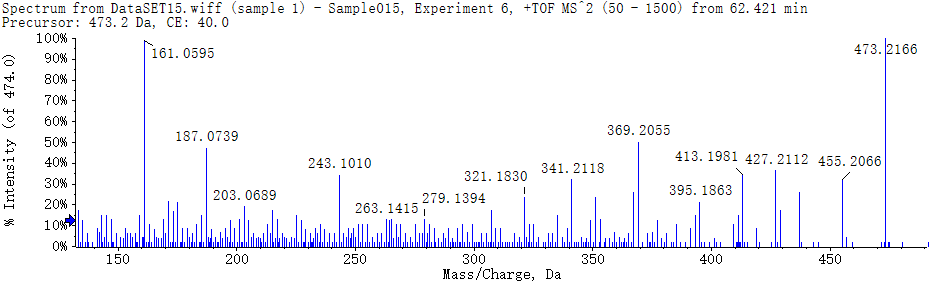


Fig. S25. Secondary mass spectrogram of component deacetylnomilin


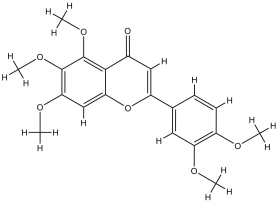

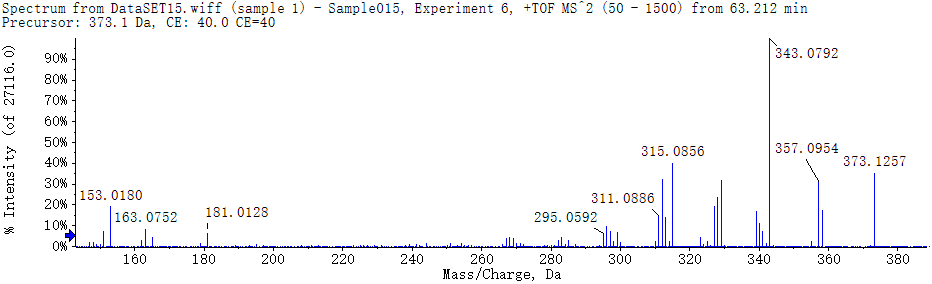


Fig. S26. Secondary mass spectrogram of component sinensetin


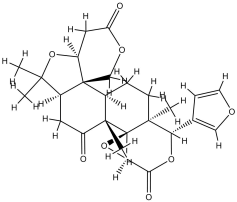

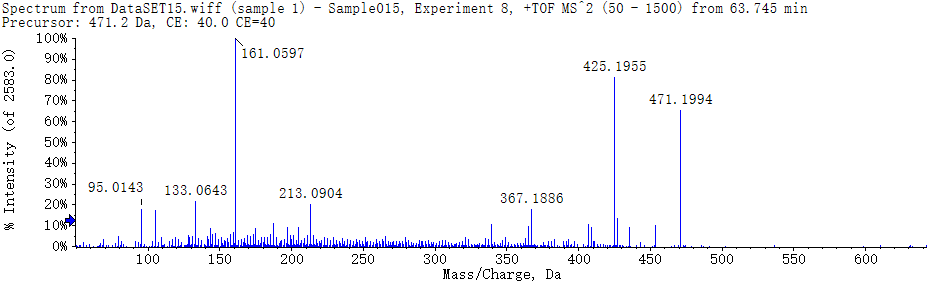


Fig. S27. Secondary mass spectrogram of component limonin


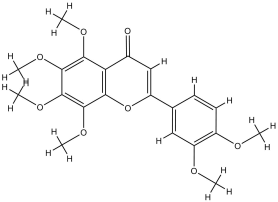

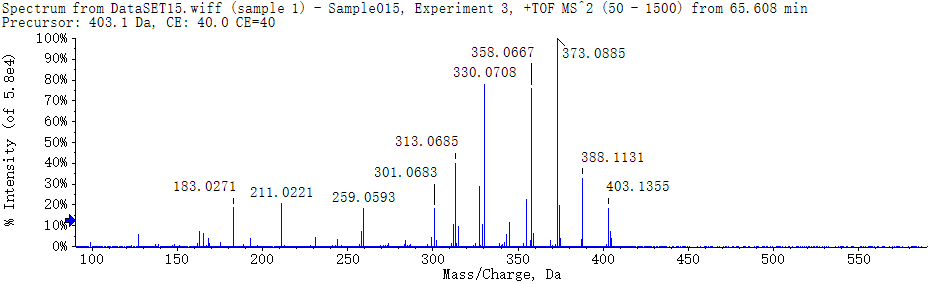


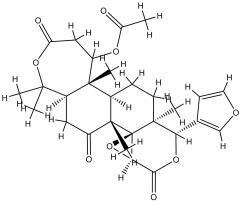
Fig. S28. Secondary mass spectrogram of component nobiletin


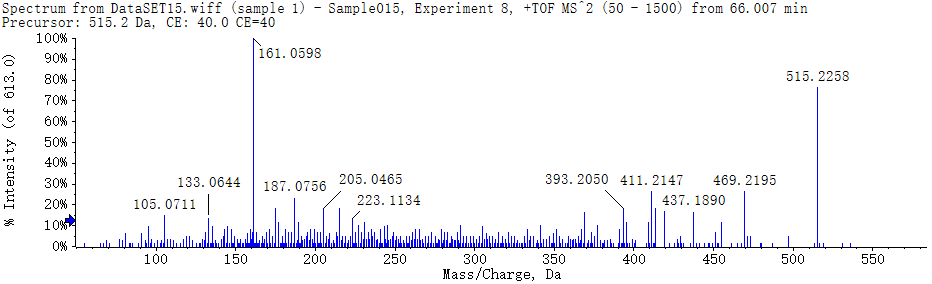


Fig. S29. Secondary mass spectrogram of component nomilin


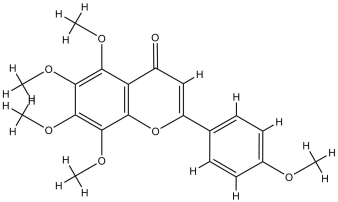

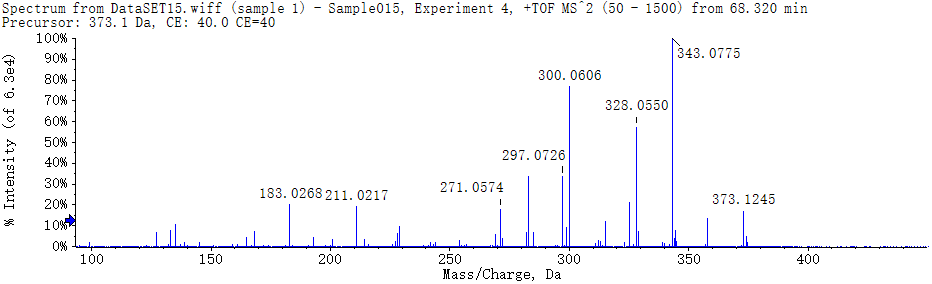


Fig. S30. Secondary mass spectrogram of component tangeretin


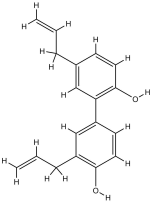


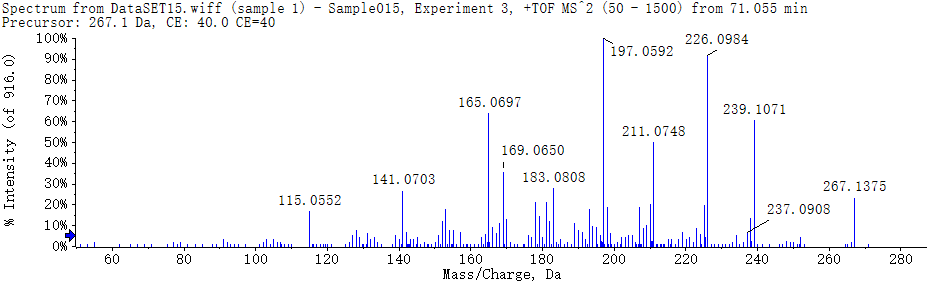


Fig. S31. Secondary mass spectrogram of component honokiol


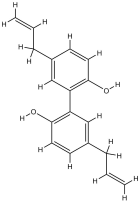


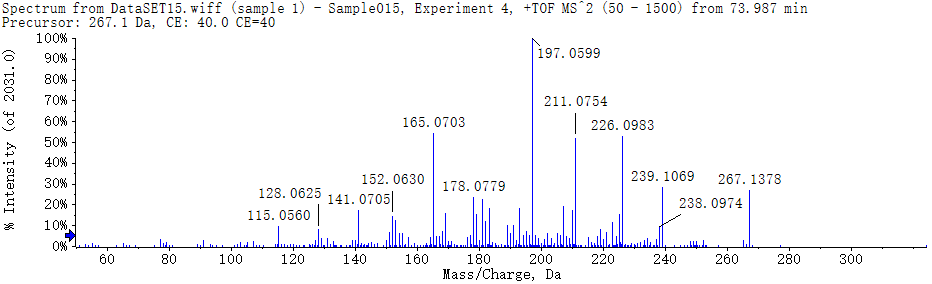


Fig. S32. Secondary mass spectrogram of component magnolol





Fig. S33 Typical HPLC chromatograms (detection wavelength 254 nm) of the seven components in ethanol extract of ZHD A. Mixed standard solution B. Sample solution

1. Genipin 1-gentiobioside; 2. geniposide; 3. naringin; 4.hesperidin; 5. neohesperidin; 6. honokiol; 7.magnolol.
