# Supplementary material for: Integrated Network Pharmacology Analysis and Experimental Validation to Investigate the Mechanism of Zhi-Zi-Hou-Po Decoction in Depression
Source: Front Pharmacol. 2021 Oct 8;12:711303. doi: 10.3389/fphar.2021.711303 (PMC8531485; doi:10.3389/fphar.2021.711303)
Supplement: Supplementary file 2 [file DataSheet1.docx]

Table S1 The target genes of thirty-one compound

| **Compound** | **Targets** | **Compound** | **Targets** |
| --- | --- | --- | --- |
| hesperidin | CYP19A1 | nobiletin | ABCG2 |
| hesperidin | SRD5A1 | nobiletin | AKR1B1 |
| hesperidin | SLC5A1 | nobiletin | CYP1B1 |
| hesperidin | SLC5A2 | nobiletin | OPRD1 |
| hesperidin | CA12 | nobiletin | KIT |
| hesperidin | CA2 | nobiletin | OPRM1 |
| hesperidin | CA1 | nobiletin | NOX4 |
| hesperidin | PTGS1 | nobiletin | CA2 |
| hesperidin | TYR | nobiletin | ALOX5 |
| hesperidin | EPHX2 | nobiletin | NOS2 |
| hesperidin | CYP1B1 | nobiletin | PTGS2 |
| hesperidin | SLC5A4 | nobiletin | ADORA1 |
| hesperidin | MMP13 | nobiletin | ADORA2A |
| hesperidin | MMP12 | nobiletin | ADORA3 |
| hesperidin | CHIA | poncirin | CYP19A1 |
| hesperidin | CA3 | poncirin | SLC5A2 |
| hesperidin | CA6 | poncirin | SLC5A1 |
| hesperidin | CA13 | poncirin | PTGS1 |
| hesperidin | CA5B | poncirin | SLC28A3 |
| hesperidin | ABCB1 | poncirin | SRD5A1 |
| hesperidin | CA5A | poncirin | EIF4A1 |
| hesperidin | CA4 | poncirin | LGALS7 |
| hesperidin | ADORA1 | poncirin | CA12 |
| hesperidin | TAS2R31 | poncirin | CA14 |
| hesperidin | CA14 | poncirin | SLC5A4 |
| hesperidin | ADORA2A | poncirin | CA2 |
| hesperidin | CA7 | poncirin | CA1 |
| hesperidin | ADORA2B | poncirin | LGALS1 |
| hesperidin | MMP7 | poncirin | ADORA1 |
| hesperidin | MMP8 | poncirin | NADK |
| hesperidin | IMPDH1 | poncirin | ADORA3 |
| hesperidin | SLC29A1 | poncirin | TAS2R31 |
| hesperidin | TERT | poncirin | FHIT |
| hesperidin | ADORA3 | poncirin | MAOB |
| bergapten | CBR1 | poncirin | CYP1B1 |
| bergapten | KCNA5 | poncirin | ABCG2 |
| bergapten | KCNA3 | poncirin | MGMT |
| bergapten | CA12 | poncirin | EPHX2 |
| bergapten | CA9 | poncirin | VARS |
| bergapten | CA7 | poncirin | LARS |
| bergapten | CA13 | poncirin | TYR |
| bergapten | CA1 | poncirin | ADORA2A |
| bergapten | ALOX5 | poncirin | ABCC1 |
| bergapten | ACHE | poncirin | HSD17B1 |
| bergapten | CYP1A2 | poncirin | SHBG |
| bergapten | CA6 | poncirin | CBR1 |
| bergapten | CA14 | poncirin | IMPDH1 |
| bergapten | CA4 | honokiol | ALOX5 |
| bergapten | CA5A | honokiol | CNR1 |
| bergapten | XDH | honokiol | CNR2 |
| bergapten | SRD5A1 | honokiol | CA2 |
| bergapten | CA5B | honokiol | KCNMA1 |
| bergapten | AKR1C3 | honokiol | MAOB |
| bergapten | AKR1C1 | honokiol | TRPV1 |
| bergapten | BACE1 | honokiol | PDK1 |
| bergapten | PARP1 | honokiol | COMT |
| bergapten | PARP2 | honokiol | HDAC8 |
| bergapten | ESR2 | honokiol | CETP |
| bergapten | MAOA | Honokiol | HSP90AA1 |
| bergapten | NFKB1 | honokiol | NQO2 |
| bergapten | GPR35 | honokiol | NR3C1 |
| bergapten | AKR1B1 | honokiol | PLA2G2A |
| bergapten | CCND1 | honokiol | ERBB2 |
| bergapten | PDGFRB | honokiol | EGFR |
| bergapten | FLT4 | honokiol | HSP90AB1 |
| bergapten | INSR | honokiol | STS |
| bergapten | PTK2 | honokiol | GABRA1 |
| bergapten | KDR | honokiol | PARP1 |
| bergapten | PLK1 | honokiol | UQCRB |
| bergapten | MET | honokiol | OPRM1 |
| bergapten | PLK4 | honokiol | OPRD1 |
| bergapten | TEK | honokiol | ALOX15 |
| bergapten | MAP3K8 | honokiol | EPHX2 |
| bergapten | BRAF | honokiol | HDAC6 |
| bergapten | EPHB4 | honokiol | MIF |
| bergapten | HSPA1A | honokiol | MAPK14 |
| bergapten | NUAK1 | honokiol | FAAH |
| bergapten | SQLE | honokiol | CES1 |
| bergapten | FGR | honokiol | DYRK1A |
| bergapten | LYN | honokiol | KIF11 |
| magnolol | CNR1 | honokiol | GSK3B |
| magnolol | CNR2 | honokiol | GSK3A |
| magnolol | ALOX5 | honokiol | TTR |
| magnolol | CA2 | honokiol | CA1 |
| magnolol | ERBB2 | honokiol | CA9 |
| magnolol | EGFR | honokiol | PAK1 |
| magnolol | DYRK1A | honokiol | ALOX5AP |
| magnolol | MIF | honokiol | NOS1 |
| magnolol | CDK5 | honokiol | CDC25B |
| magnolol | CCNB3 | honokiol | ADORA2A |
| magnolol | CDK1 | honokiol | AURKB |
| magnolol | CCNB1 | honokiol | SRC |
| magnolol | CCNB2 | honokiol | PTK2 |
| magnolol | ADORA1 | honokiol | KDR |
| magnolol | OPRD1 | honokiol | MET |
| magnolol | EPHX2 | honokiol | ALK |
| magnolol | ADORA2A | honokiol | NEK6 |
| magnolol | JAK2 | honokiol | TRAP1 |
| magnolol | MDM2 | honokiol | NOX4 |
| magnolol | KDR | honokiol | NOX1 |
| magnolol | HSD11B1 | honokiol | GRIN1 |
| magnolol | CXCR2 | honokiol | NR1H4 |
| magnolol | RCE1 | honokiol | MMP9 |
| magnolol | MAOB | honokiol | CA12 |
| magnolol | AURKB | honokiol | MMP1 |
| magnolol | AURKC | honokiol | MMP2 |
| magnolol | PAK1 | honokiol | PRKCG |
| magnolol | AURKA | honokiol | HSD17B1 |
| magnolol | HDAC8 | honokiol | BRAF |
| magnolol | GPR55 | honokiol | PTGER4 |
| magnolol | CETP | honokiol | MGLL |
| magnolol | ALOX5AP | honokiol | HSD11B1 |
| magnolol | GSK3B | honokiol | CCNE1 |
| magnolol | ABCG2 | honokiol | CDK2 |
| magnolol | MC4R | honokiol | FLT3 |
| magnolol | KCNMA1 | honokiol | PDE4B |
| magnolol | AKR1C3 | honokiol | NISCH |
| magnolol | TYMS | honokiol | SIRT2 |
| magnolol | TRPV1 | honokiol | GPBAR1 |
| magnolol | CA12 | honokiol | CYP11B1 |
| magnolol | STS | honokiol | CYP19A1 |
| magnolol | CA9 | honokiol | ACHE |
| magnolol | CA1 | honokiol | CYP11B2 |
| magnolol | ADRA2A | honokiol | SHBG |
| magnolol | ADRA2B | honokiol | DYRK1B |
| magnolol | ADRA1A | honokiol | RET |
| magnolol | ALOX15 | honokiol | PGR |
| magnolol | DYRK2 | honokiol | HDAC1 |
| magnolol | CPB1 | honokiol | FGFR1 |
| magnolol | FGFR1 | honokiol | CSF1R |
| magnolol | EGLN1 | honokiol | PIM1 |
| magnolol | OPRM1 | honokiol | PDPK1 |
| magnolol | MMP9 | honokiol | PTGES |
| magnolol | MMP1 | honokiol | ATP4B |
| magnolol | MMP2 | honokiol | ADRA2A |
| magnolol | HDAC6 | honokiol | UPP1 |
| magnolol | PDE4B | honokiol | HSD17B2 |
| magnolol | HSD17B3 | honokiol | FLT1 |
| magnolol | HSD17B2 | honokiol | PDGFRB |
| magnolol | PPP1CA | honokiol | KIT |
| magnolol | PARP1 | honokiol | SGK1 |
| magnolol | TACR3 | honokiol | CLK1 |
| magnolol | CSF1R | honokiol | CDC7 |
| magnolol | RAF1 | honokiol | RAF1 |
| magnolol | BRAF | honokiol | GPR84 |
| magnolol | ADAMTS5 | honokiol | TYR |
| magnolol | P2RY1 | honokiol | PRKCZ |
| magnolol | NISCH | honokiol | ALOX5 |
| magnolol | FLT3 | feretoside | MGAM |
| magnolol | CDK5R1 | feretoside | AKR1B1 |
| magnolol | ADRA2C | feretoside | CA2 |
| magnolol | RET | feretoside | CA1 |
| magnolol | HTR1A | feretoside | CA12 |
| magnolol | MAPK14 | feretoside | CA9 |
| magnolol | NQO1 | feretoside | IMPDH1 |
| magnolol | NQO2 | feretoside | IMPDH2 |
| magnolol | RIPK2 | feretoside | SI |
| magnolol | PANK3 | feretoside | PNP |
| magnolol | PTGES | feretoside | BCL2L1 |
| magnolol | TUBB1 | feretoside | ADORA1 |
| magnolol | CYP19A1 | feretoside | ADORA2A |
| magnolol | SIRT2 | feretoside | ADORA3 |
| magnolol | CHEK1 | feretoside | ADA |
| magnolol | MYLK | feretoside | P2RX3 |
| magnolol | HDAC1 | feretoside | TYMP |
| magnolol | CCNE1 | genipin | PTGS1 |
| magnolol | CDK2 | genipin | PTGS2 |
| magnolol | ALOX12 | genipin | CA2 |
| magnolol | CHRM1 | genipin | GABRA2 |
| magnolol | AKT1 | genipin | GABRA1 |
| magnolol | TNNI3K | genipin | TK1 |
| magnolol | UQCRB | genipin | PRSS1 |
| magnolol | ADORA3 | genipin | GRIA2 |
| magnolol | PTK2 | genipin | GABRA6 |
| magnolol | PLK1 | genipin 1-gentiobioside | CA2 |
| magnolol | MGLL | genipin 1-gentiobioside | CA1 |
| magnolol | KDM4E | genipin 1-gentiobioside | CA12 |
| magnolol | ADRA1D | genipin 1-gentiobioside | CA9 |
| magnolol | ADRA1B | genipin 1-gentiobioside | TYR |
| magnolol | HSP90AB1 | genipin 1-gentiobioside | ADORA2A |
| magnolol | ASAH1 | genipin 1-gentiobioside | CHIA |
| magnolol | IDH1 | genipin 1-gentiobioside | AMY2A |
| geniposidic acid | ADORA1 | genipin 1-gentiobioside | CA14 |
| geniposidic acid | SELP | genipin 1-gentiobioside | AMY1A |
| geniposidic acid | ENGASE | genipin 1-gentiobioside | GAA |
| geniposidic acid | YARS | genipin 1-gentiobioside | ADORA1 |
| geniposidic acid | MGAM | genipin 1-gentiobioside | ADORA3 |
| geniposidic acid | OGA | genipin 1-gentiobioside | ADORA2B |
| geniposidic acid | NAALAD2 | genipin 1-gentiobioside | EPHX2 |
| geniposidic acid | NEU3 | tangeretin | OPRD1 |
| geniposidic acid | NEU2 | tangeretin | AKR1B1 |
| geniposidic acid | FOLH1 | tangeretin | OPRM1 |
| geniposidic acid | BCL2L1 | tangeretin | ABCG2 |
| geniposidic acid | AMY1A | tangeretin | KIT |
| geniposidic acid | GAA | tangeretin | CYP1B1 |
| geniposidic acid | NEU4 | tangeretin | NOS2 |
| geniposidic acid | SELL | tangeretin | PTGS2 |
| caffeic acid | ESR1 | tangeretin | ADORA3 |
| caffeic acid | SLC6A2 | tangeretin | ADORA1 |
| caffeic acid | MAPK1 | tangeretin | ADORA2A |
| caffeic acid | AKR1C4 | tangeretin | PFKFB3 |
| caffeic acid | TPMT | tangeretin | TERT |
| caffeic acid | NGFR | tangeretin | ALOX5 |
| caffeic acid | SYK | tangeretin | ABCB1 |
| caffeic acid | FYN | tangeretin | PLG |
| caffeic acid | LCK | tangeretin | FLT3 |
| caffeic acid | APP | tangeretin | PIM1 |
| caffeic acid | KDM4E | tangeretin | PTPRS |
| caffeic acid | KDM4A | tangeretin | MMP13 |
| caffeic acid | EGFR | tangeretin | NOX4 |
| caffeic acid | CTBP2 | tangeretin | IGF1R |
| caffeic acid | MAOB | tangeretin | APP |
| caffeic acid | PTGS1 | tangeretin | AURKB |
| caffeic acid | PIK3CB | tangeretin | KDR |
| caffeic acid | CYP1A2 | tangeretin | CYP1A1 |
| caffeic acid | CYP2C9 | tangeretin | CYP19A1 |
| caffeic acid | CYP3A4 | tangeretin | AMY1A |
| caffeic acid | CYP2C19 | tangeretin | GRK6 |
| caffeic acid | PIK3CA | tangeretin | MCL1 |
| caffeic acid | KDM3A | tangeretin | SYK |
| caffeic acid | KDM6B | tangeretin | EGFR |
| caffeic acid | FTO | tangeretin | TYR |
| caffeic acid | KDM4C | tangeretin | AHR |
| caffeic acid | ELANE | tangeretin | ESRRA |
| caffeic acid | MMP8 | tangeretin | PLK1 |
| caffeic acid | F3 | tangeretin | MET |
| caffeic acid | HSD11B1 | tangeretin | ACHE |
| caffeic acid | NFE2L2 | tangeretin | CA2 |
| caffeic acid | STAT3 | tangeretin | CA12 |
| rutin | NMUR2 | tangeretin | F2 |
| rutin | ADRA2A | tangeretin | SRC |
| rutin | ADRA2C | tangeretin | TNKS2 |
| rutin | ACHE | tangeretin | TNKS |
| rutin | AKR1B1 | tangeretin | IKBKB |
| rutin | CA7 | tangeretin | GPR35 |
| rutin | CA12 | tangeretin | DAPK1 |
| rutin | CA4 | tangeretin | MPG |
| rutin | NOX4 | tangeretin | HSD17B2 |
| rutin | CA2 | tangeretin | HSD17B1 |
| rutin | RPS6KA3 | tangeretin | PTK2 |
| rutin | NQO2 | tangeretin | MMP9 |
| rutin | XDH | tangeretin | ALOX12 |
| rutin | CD38 | tangeretin | INSR |
| rutin | PTGS2 | tangeretin | ALK |
| rutin | PDE5A | tangeretin | NTRK2 |
| rutin | TNF | tangeretin | CSNK2A1 |
| rutin | IL2 | tangeretin | PYGL |
| rutin | ADORA1 | tangeretin | GSK3B |
| rutin | ALOX5 | tangeretin | PARP1 |
| rutin | VCP | tangeretin | GLO1 |
| rutin | TNNT2 | tangeretin | MPO |
| rutin | TNNI3K | tangeretin | PIK3R1 |
| rutin | TNNC1 | tangeretin | CA3 |
| rutin | ADORA3 | tangeretin | PKN1 |
| rutin | SQLE | tangeretin | NEK2 |
| rutin | SLC29A1 | tangeretin | CXCR1 |
| rutin | PLG | tangeretin | NEK6 |
| rutin | TP53 | tangeretin | APEX1 |
| rutin | ABCG2 | tangeretin | NUAK1 |
| rutin | CYP1B1 | tangeretin | AKR1C2 |
| rutin | KCNA3 | tangeretin | AKR1C1 |
| rutin | TERT | tangeretin | AKR1C4 |
| chlorogenic acid | AKR1B1 | tangeretin | AKR1A1 |
| chlorogenic acid | AKR1B10 | tangeretin | ESR2 |
| chlorogenic acid | MMP12 | tangeretin | DRD4 |
| chlorogenic acid | APP | tangeretin | MMP2 |
| chlorogenic acid | MMP13 | tangeretin | CDK5 |
| chlorogenic acid | MMP2 | tangeretin | CDK5R1 |
| chlorogenic acid | SLC37A4 | tangeretin | CDK1 |
| chlorogenic acid | PYGL | tangeretin | CCNB1 |
| chlorogenic acid | PRKCD | tangeretin | CCNB2 |
| chlorogenic acid | PRKCA | tangeretin | CCNB3 |
| chlorogenic acid | CA2 | tangeretin | CYP1A2 |
| chlorogenic acid | CA1 | tangeretin | CA1 |
| chlorogenic acid | CA12 | tangeretin | CA9 |
| chlorogenic acid | CA9 | tangeretin | ODC1 |
| chlorogenic acid | NEU4 | tangeretin | TOP1 |
| chlorogenic acid | BACE1 | tangeretin | ARG1 |
| chlorogenic acid | PDE4D | tangeretin | PIK3CG |
| chlorogenic acid | PDE9A | tangeretin | BMP4 |
| chlorogenic acid | PDE1B | tangeretin | ABCC1 |
| chlorogenic acid | CASP3 | tangeretin | CA7 |
| chlorogenic acid | CA5B | tangeretin | TUBB1 |
| chlorogenic acid | ABCB1 | tangeretin | AGPAT2 |
| chlorogenic acid | NEU3 | tangeretin | PRKAB1 |
| chlorogenic acid | NEU2 | tangeretin | BMPR1A |
| chlorogenic acid | ENGASE | tangeretin | ACVR1B |
| chlorogenic acid | ECE1 | tangeretin | XDH |
| chlorogenic acid | OGA | tangeretin | MMP3 |
| chlorogenic acid | ELANE | tangeretin | BACE1 |
| chlorogenic acid | KDR | tangeretin | CAMK2B |
| chlorogenic acid | CASP6 | tangeretin | MAOA |
| chlorogenic acid | CASP7 | nomilin | OPRK1 |
| chlorogenic acid | CASP8 | nomilin | OPRD1 |
| chlorogenic acid | CASP1 | nomilin | OPRM1 |
| chlorogenic acid | CASP2 | nomilin | C5AR1 |
| chlorogenic acid | EGLN1 | nomilin | MTOR |
| chlorogenic acid | ADAMTS5 | nomilin | PIK3CA |
| chlorogenic acid | FTO | nomilin | CTSK |
| chlorogenic acid | DNMT3B | nomilin | CTSS |
| chlorogenic acid | TREH | nomilin | CTSV |
| limonin | OPRD1 | nomilin | CYP51A1 |
| limonin | OPRK1 | nomilin | MAPK1 |
| limonin | OPRM1 | nomilin | TNKS2 |
| limonin | CTSK | nomilin | CNR1 |
| limonin | CTSS | nomilin | CNR2 |
| limonin | LRRK2 | nomilin | DGAT1 |
| limonin | P2RX3 | nomilin | CTSL |
| limonin | MAPK1 | nomilin | PDE10A |
| limonin | BACE1 | nomilin | TRPV1 |
| limonin | PSMB8 | nomilin | P2RX3 |
| limonin | TERT | nomilin | FASN |
| limonin | CDK9 | nomilin | HSD11B1 |
| limonin | CCR1 | nomilin | CHRM3 |
| limonin | CTSL | nomilin | TAAR1 |
| limonin | CCNE1 | nomilin | CSF1R |
| limonin | CDK2 | nomilin | BRD4 |
| limonin | C5AR1 | nomilin | BRD3 |
| limonin | DRD2 | nomilin | PARP1 |
| limonin | GSK3B | nomilin | PTPN1 |
| limonin | GSK3A | nomilin | MAP2K1 |
| limonin | P2RX7 | nomilin | MMP13 |
| limonin | FKBP1A | nomilin | MMP2 |
| limonin | MTOR | nomilin | LIPE |
| limonin | PIK3CA | nomilin | LRRK2 |
| limonin | PDGFRB | nomilin | PTGER1 |
| limonin | KIT | nomilin | PTGER4 |
| limonin | KDR | nomilin | PSENEN |
| limonin | HDAC1 | nomilin | NCSTN |
| limonin | HDAC4 | nomilin | APH1A |
| limonin | ITK | nomilin | PSEN1 |
| limonin | IRAK4 | nomilin | APH1B |
| limonin | NR3C1 | nomilin | PSEN2 |
| limonin | PGR | nomilin | S1PR3 |
| limonin | MMP1 | nomilin | CCNE1 |
| limonin | LIMK2 | nomilin | ADRA2B |
| limonin | CHRM3 | nomilin | DRD3 |
| limonin | CCNC | nomilin | CXCR2 |
| limonin | MDM2 | nomilin | ADORA3 |
| limonin | CDK8 | nomilin | LIMK2 |
| limonin | RHOA | nomilin | CFD |
| limonin | PSENEN | nomilin | MAPK8 |
| limonin | NCSTN | nomilin | PRCP |
| limonin | APH1A | nomilin | EPHX2 |
| limonin | PSEN1 | nomilin | F10 |
| limonin | APH1B | nomilin | HDAC1 |
| limonin | PSEN2 | nomilin | CTSB |
| limonin | EPHB4 | nomilin | NR1H4 |
| limonin | CNR1 | nomilin | AURKB |
| limonin | CNR2 | nomilin | PYGL |
| limonin | NR1I2 | nomilin | ROCK2 |
| limonin | ADRA2B | nomilin | CYP2C9 |
| limonin | DRD3 | nomilin | NR1I2 |
| limonin | ADORA3 | nomilin | CYP2C19 |
| limonin | PARP1 | nomilin | HMGCR |
| limonin | FASN | nomilin | NR5A1 |
| limonin | PSMB5 | nomilin | AURKA |
| limonin | F2 | nomilin | HPGDS |
| limonin | FNTB | nomilin | CASR |
| limonin | FNTA | nomilin | BACE2 |
| limonin | PGGT1B | nomilin | GSK3B |
| limonin | F10 | nomilin | GSK3A |
| limonin | SRC | nomilin | PDE9A |
| limonin | CTSV | nomilin | BACE1 |
| limonin | GRK7 | nomilin | NAMPT |
| limonin | STK38 | nomilin | EGFR |
| limonin | HIPK4 | nomilin | CASP8 |
| limonin | ERN1 | nomilin | CASP1 |
| limonin | OXSR1 | nomilin | MAOB |
| limonin | STK39 | nomilin | HTR2A |
| limonin | MAP3K13 | nomilin | CDK5 |
| limonin | ICK | nomilin | CCNA1 |
| limonin | MAP3K15 | nomilin | CCNA2 |
| limonin | MAST1 | nomilin | ICAM1 |
| limonin | SBK1 | nomilin | ITGB2 |
| limonin | HUNK | nomilin | CCR1 |
| limonin | FYN | nomilin | F2R |
| limonin | CSF1R | nomilin | CDK5R1 |
| limonin | ABL1 | nomilin | CDK2 |
| limonin | FLT1 | nomilin | DYRK1A |
| limonin | PRPF4B | nomilin | ADCY1 |
| limonin | SNRK | nomilin | HTR2C |
| limonin | DSTYK | nomilin | PDE2A |
| limonin | MAP3K12 | nomilin | AKR1C3 |
| limonin | FLT4 | nomilin | ITGAL |
| limonin | FLT3 | nomilin | CDK9 |
| limonin | INSR | nomilin | TNF |
| limonin | IKBKB | nomilin | KCNH2 |
| limonin | PDGFRA | nomilin | MAPK14 |
| limonin | EGFR | nomilin | AOC3 |
| limonin | RET | nomilin | BCL2L1 |
| limonin | YES1 | nomilin | LDHA |
| limonin | MAP2K3 | nomilin | ATR |
| limonin | PRKAA2 | nomilin | WNT3 |
| limonin | JAK3 | nomilin | IDH1 |
| limonin | MAP2K6 | nomilin | RET |
| limonin | AURKB | nomilin | FNTB |
| limonin | BLK | nomilin | FNTA |
| limonin | DYRK1A | nomilin | NTRK1 |
| limonin | RPS6KA3 | nomilin | JAK2 |
| limonin | PHKG2 | nomilin | PER2 |
| limonin | CSNK1G1 | nomilin | NR3C1 |
| limonin | MYLK | gardenoside | IL2 |
| limonin | DAPK3 | gardenoside | MGAM |
| limonin | CAMK1 | gardenoside | IMPDH1 |
| limonin | CAMK4 | gardenoside | IMPDH2 |
| limonin | CHEK2 | gardenoside | CA2 |
| limonin | PDPK1 | gardenoside | FUCA1 |
| limonin | OPRD1 | gardenoside | ADORA1 |
| limonin | OPRK1 | gardenoside | CA12 |
| methyl deacetylasperulosidate | MGAM | gardenoside | ADORA2A |
| methyl deacetylasperulosidate | AKR1B1 | gardenoside | HK2 |
| methyl deacetylasperulosidate | CA2 | gardenoside | HK1 |
| methyl deacetylasperulosidate | CA1 | gardenoside | CA1 |
| methyl deacetylasperulosidate | CA12 | gardenoside | LGALS3 |
| methyl deacetylasperulosidate | CA9 | gardenoside | FHIT |
| methyl deacetylasperulosidate | IMPDH1 | gardenoside | ADH1A |
| methyl deacetylasperulosidate | IMPDH2 | gardenoside | TYMP |
| methyl deacetylasperulosidate | SI | gardenoside | ADORA3 |
| methyl deacetylasperulosidate | PNP | gardenoside | PNP |
| methyl deacetylasperulosidate | BCL2L1 | gardenoside | CA9 |
| methyl deacetylasperulosidate | ADORA1 | scandoside | ADORA1 |
| methyl deacetylasperulosidate | ADORA2A | scandoside | SELP |
| methyl deacetylasperulosidate | ADORA3 | scandoside | BCL2L1 |
| methyl deacetylasperulosidate | ADA | scandoside | PGD |
| methyl deacetylasperulosidate | P2RX3 | scandoside | CA2 |
| methyl deacetylasperulosidate | TYMP | scandoside | CA1 |
| deacetylnomilin | OPRK1 | scandoside | CA12 |
| deacetylnomilin | OPRD1 | scandoside | CA9 |
| deacetylnomilin | OPRM1 | scandoside | MGAM |
| deacetylnomilin | AKR1C3 | scandoside | HPRT1 |
| deacetylnomilin | PER2 | scandoside | FGF2 |
| deacetylnomilin | ALOX5 | scandoside | P2RY1 |
| deacetylnomilin | CCR1 | scandoside | YARS |
| deacetylnomilin | BRD4 | shanzhiside methyl ester | IMPDH1 |
| deacetylnomilin | CREBBP | shanzhiside methyl ester | IMPDH2 |
| deacetylnomilin | PSENEN | shanzhiside methyl ester | CA2 |
| deacetylnomilin | NCSTN | shanzhiside methyl ester | TYMP |
| deacetylnomilin | APH1A | shanzhiside methyl ester | MGAM |
| deacetylnomilin | PSEN1 | shanzhiside methyl ester | SI |
| deacetylnomilin | APH1B | shanzhiside methyl ester | AKR1B1 |
| deacetylnomilin | PSEN2 | shanzhiside methyl ester | FUCA1 |
| deacetylnomilin | TERT | shanzhiside methyl ester | TYR |
| deacetylnomilin | NR1I2 | shanzhiside methyl ester | ADORA1 |
| deacetylnomilin | P2RX3 | shanzhiside methyl ester | CA1 |
| deacetylnomilin | MAPK1 | shanzhiside methyl ester | CA12 |
| deacetylnomilin | AR | shanzhiside methyl ester | CA14 |
| deacetylnomilin | NR3C1 | shanzhiside methyl ester | CA9 |
| deacetylnomilin | F10 | shanzhiside methyl ester | ADORA2A |
| deacetylnomilin | MMP9 | caryoptoside | ADORA1 |
| deacetylnomilin | MET | caryoptoside | ADORA2A |
| deacetylnomilin | LRRK2 | caryoptoside | CA2 |
| deacetylnomilin | MAPK8 | caryoptoside | CA1 |
| deacetylnomilin | CNR2 | caryoptoside | CA12 |
| deacetylnomilin | MAPK10 | caryoptoside | CA14 |
| deacetylnomilin | CES2 | caryoptoside | CA9 |
| deacetylnomilin | ADAM17 | caryoptoside | SI |
| deacetylnomilin | MAPK9 | caryoptoside | FUCA1 |
| deacetylnomilin | TYRO3 | caryoptoside | TYR |
| deacetylnomilin | MERTK | caryoptoside | MGAM |
| deacetylnomilin | PIK3CA | caryoptoside | LGALS3 |
| deacetylnomilin | CCNC | caryoptoside | LGALS9 |
| deacetylnomilin | CDK8 | caryoptoside | ADA |
| deacetylnomilin | IMPDH1 | caryoptoside | ADORA3 |
| deacetylnomilin | IMPDH2 | caryoptoside | EPHX2 |
| deacetylnomilin | HSD17B2 | caryoptoside | SLC6A2 |
| deacetylnomilin | HSD11B1 | caryoptoside | FOLH1 |
| deacetylnomilin | SORD | caryoptoside | AKR1C3 |
| deacetylnomilin | MMP1 | caryoptoside | HPRT1 |
| deacetylnomilin | MMP2 | caryoptoside | HK2 |
| deacetylnomilin | MMP8 | caryoptoside | HK1 |
| deacetylnomilin | TRAP1 | caryoptoside | CA7 |
| deacetylnomilin | HSP90AB1 | caryoptoside | CA4 |
| deacetylnomilin | MAPK14 | caryoptoside | CA13 |
| deacetylnomilin | MMP3 | caryoptoside | CA5A |
| deacetylnomilin | GSK3B | caryoptoside | ADK |
| deacetylnomilin | CDK1 | caryoptoside | SLC5A2 |
| deacetylnomilin | CCNB1 | caryoptoside | TYMP |
| deacetylnomilin | CCNB2 | caryoptoside | HSP90AA1 |
| deacetylnomilin | CCNA1 | caryoptoside | CA6 |
| deacetylnomilin | CCNA2 | caryoptoside | PNP |
| deacetylnomilin | MTOR | caryoptoside | IGFBP3 |
| deacetylnomilin | PDE5A | caryoptoside | CDA |
| deacetylnomilin | CCNB3 | caryoptoside | TREH |
| deacetylnomilin | RPS6KA3 | caryoptoside | AMY2A |
| deacetylnomilin | BACE2 | caryoptoside | ATIC |
| deacetylnomilin | BACE1 | caryoptoside | ALOX12 |
| deacetylnomilin | TTL | sinensetin | ABCG2 |
| deacetylnomilin | FAAH | sinensetin | CYP1B1 |
| deacetylnomilin | NTRK1 | sinensetin | ADORA1 |
| deacetylnomilin | ABCB1 | sinensetin | ADORA2A |
| deacetylnomilin | GABRB3 | sinensetin | AKR1B1 |
| deacetylnomilin | CHRM1 | sinensetin | OPRD1 |
| deacetylnomilin | PARP1 | sinensetin | PLG |
| deacetylnomilin | PDK1 | sinensetin | ADORA3 |
| deacetylnomilin | FKBP1A | sinensetin | KIT |
| deacetylnomilin | ITK | sinensetin | FLT3 |
| deacetylnomilin | PIK3CD | sinensetin | CA2 |
| deacetylnomilin | PIK3CB | sinensetin | CA7 |
| deacetylnomilin | CHEK1 | sinensetin | GSK3B |
| deacetylnomilin | AURKA | sinensetin | SYK |
| deacetylnomilin | CCND1 | sinensetin | NOX4 |
| deacetylnomilin | CDK4 | sinensetin | ALOX5 |
| deacetylnomilin | ICAM1 | sinensetin | ABCB1 |
| deacetylnomilin | ITGB2 | sinensetin | PIM1 |
| deacetylnomilin | KIT | sinensetin | PTPRS |
| deacetylnomilin | KDR | sinensetin | CDK5 |
| deacetylnomilin | P2RX7 | sinensetin | CDK5R1 |
| deacetylnomilin | GSK3A | sinensetin | GLO1 |
| deacetylnomilin | CFTR | sinensetin | AKR1B10 |
| deacetylnomilin | CCNE2 | sinensetin | TOP1 |
| deacetylnomilin | MMP13 | sinensetin | ARG1 |
| deacetylnomilin | CDK2 | sinensetin | ABCC1 |
| deacetylnomilin | GYS1 | sinensetin | CA12 |
| deacetylnomilin | WNT3A | sinensetin | TNKS |
| deacetylnomilin | ITGAL | sinensetin | PARP1 |
| deacetylnomilin | HMGCR | sinensetin | TNKS2 |
| deacetylnomilin | LIMK2 | sinensetin | AMY1A |
| deacetylnomilin | TNKS2 | sinensetin | GRK6 |
| deacetylnomilin | PAK1 | sinensetin | PLK1 |
| deacetylnomilin | CSF1R | sinensetin | MET |
| deacetylnomilin | FLT4 | sinensetin | MMP2 |
| deacetylnomilin | FLT3 | sinensetin | MAOA |
| deacetylnomilin | JAK3 | sinensetin | CCNB3 |
| deacetylnomilin | ADCY1 | sinensetin | CCNB1 |
| deacetylnomilin | PIK3CG | sinensetin | CCNB2 |
| deacetylnomilin | PDE10A | sinensetin | APP |
| deacetylnomilin | KCNJ1 | sinensetin | CA1 |
| deacetylnomilin | PRKCZ | sinensetin | CA13 |
| deacetylnomilin | PGR | sinensetin | CDK1 |
| deacetylnomilin | ADORA2A | sinensetin | KDR |
| deacetylnomilin | MAP2K1 | sinensetin | ESR2 |
| deacetylnomilin | TNF | sinensetin | KDM4E |
| deacetylnomilin | CDK9 | sinensetin | ALOX12 |
| deacetylnomilin | AKT1 | sinensetin | EGFR |
| deacetylnomilin | CCNE1 | sinensetin | PTGS2 |
| deacetylnomilin | PYGL | sinensetin | OPRM1 |
| deacetylnomilin | PRKDC | sinensetin | CSNK2A1 |
| deacetylnomilin | CASP8 | sinensetin | SRC |
| synephrine | NMUR2 | sinensetin | ACHE |
| synephrine | ADRA1A | sinensetin | TTR |
| synephrine | ADRB1 | sinensetin | TERT |
| synephrine | ADRA1B | sinensetin | MMP12 |
| synephrine | ADRA2A | sinensetin | PIK3CG |
| synephrine | ADRA2B | sinensetin | TOP2A |
| synephrine | ADRB2 | sinensetin | MPO |
| synephrine | ADRA2C | sinensetin | PIK3R1 |
| synephrine | DRD2 | sinensetin | CA3 |
| synephrine | ADRA1D | sinensetin | CXCR1 |
| synephrine | OPRM1 | sinensetin | APEX1 |
| synephrine | HTR3A | sinensetin | AKR1C2 |
| synephrine | ADRB3 | sinensetin | AKR1C1 |
| synephrine | DRD4 | sinensetin | AKR1C4 |
| synephrine | DRD3 | sinensetin | AKR1A1 |
| synephrine | SLC6A4 | sinensetin | XDH |
| synephrine | HTR1A | sinensetin | AURKB |
| synephrine | ANPEP | sinensetin | MMP13 |
| synephrine | MTNR1A | sinensetin | MMP9 |
| synephrine | PNP | sinensetin | BACE1 |
| synephrine | SLC6A3 | sinensetin | CA6 |
| synephrine | SLC6A2 | sinensetin | CDK2 |
| synephrine | GSR | sinensetin | ST6GAL1 |
| synephrine | GRIN2B | sinensetin | CBR1 |
| synephrine | GRIN1 | sinensetin | CD38 |
| synephrine | KCNH2 | sinensetin | PLA2G2A |
| synephrine | EBP | sinensetin | PFKFB3 |
| synephrine | DPP4 | sinensetin | CA4 |
| synephrine | HTR2A | sinensetin | CYP19A1 |
| synephrine | FUCA1 | sinensetin | DRD4 |
| synephrine | TAAR1 | sinensetin | IGF1R |
| neohesperidin | CYP19A1 | sinensetin | CAMK2B |
| neohesperidin | SRD5A1 | sinensetin | AXL |
| neohesperidin | SLC5A4 | sinensetin | PTK2 |
| neohesperidin | SLC5A2 | sinensetin | CA9 |
| neohesperidin | PTGS1 | sinensetin | NOS2 |
| neohesperidin | SLC5A1 | sinensetin | F2 |
| neohesperidin | SLC28A3 | sinensetin | CFTR |
| neohesperidin | CA2 | sinensetin | NEK2 |
| neohesperidin | CA1 | sinensetin | ALK |
| neohesperidin | CA3 | sinensetin | TYR |
| neohesperidin | CA6 | sinensetin | AHR |
| neohesperidin | CA12 | sinensetin | MAPK1 |
| neohesperidin | CA13 | sinensetin | INSR |
| neohesperidin | CA5B | sinensetin | NAE1 |
| neohesperidin | ABCB1 | sinensetin | HSD17B2 |
| neohesperidin | CA5A | sinensetin | BMP4 |
| neohesperidin | CYP1B1 | sinensetin | HSD17B1 |
| neohesperidin | CA7 | sinensetin | ESR1 |
| neohesperidin | CA4 | sinensetin | LCK |
| neohesperidin | EPHX2 | sinensetin | BCL2L1 |
| neohesperidin | MMP12 | rhoifolin | TNF |
| neohesperidin | TAS2R31 | rhoifolin | IL2 |
| neohesperidin | ADORA2A | rhoifolin | ADORA1 |
| neohesperidin | TYR | rhoifolin | AKR1B1 |
| neohesperidin | ADORA1 | rhoifolin | XDH |
| neohesperidin | EIF4A1 | rhoifolin | NMUR2 |
| isosinensetin | ABCG2 | rhoifolin | ADRA2A |
| isosinensetin | AKR1B1 | rhoifolin | ADRA2C |
| isosinensetin | OPRD1 | rhoifolin | ACHE |
| isosinensetin | OPRM1 | rhoifolin | CA2 |
| isosinensetin | KIT | rhoifolin | CA12 |
| isosinensetin | ALOX5 | rhoifolin | RPS6KA3 |
| isosinensetin | CYP1B1 | rhoifolin | NQO2 |
| isosinensetin | PTGS2 | rhoifolin | NOX4 |
| isosinensetin | ADORA3 | rhoifolin | CA7 |
| isosinensetin | NOS2 | rhoifolin | CA4 |
| isosinensetin | CA2 | rhoifolin | ALDH2 |
| isosinensetin | CA12 | rhoifolin | CA1 |
| isosinensetin | CA7 | rhoifolin | CA9 |
| isosinensetin | ABCC1 | rhoifolin | EGFR |
| isosinensetin | ADORA1 | rhoifolin | HSP90AA1 |
| isosinensetin | TERT | rhoifolin | MMP1 |
| isosinensetin | CA4 | rhoifolin | MMP7 |
| isosinensetin | IGF1R | rhoifolin | MMP8 |
| isosinensetin | INSR | rhoifolin | CD38 |
| isosinensetin | ALK | rhoifolin | IMPDH1 |
| isosinensetin | NOX4 | rhoifolin | IMPDH2 |
| isosinensetin | ADORA2A | naringin | CYP19A1 |
| isosinensetin | SYK | naringin | SLC5A1 |
| isosinensetin | MET | naringin | SLC5A4 |
| isosinensetin | CSNK2A1 | naringin | SLC5A2 |
| isosinensetin | CDK5R1 | naringin | SRD5A1 |
| isosinensetin | CDK5 | naringin | PTGS1 |
| isosinensetin | ABCB1 | naringin | EPHX2 |
| isosinensetin | EGFR | naringin | TYR |
| isosinensetin | PLK1 | naringin | ADORA1 |
| isosinensetin | AURKB | naringin | SLC28A3 |
| isosinensetin | KDR | naringin | MMP1 |
| isosinensetin | FLT3 | naringin | MMP7 |
| isosinensetin | APP | naringin | MMP8 |
| isosinensetin | GPR35 | naringin | ADORA2A |
| isosinensetin | PARP1 | naringin | ALDH2 |
| isosinensetin | SRC | naringin | MMP13 |
| isosinensetin | PIM1 | naringin | MMP12 |
| isosinensetin | F2 | naringin | TAS2R31 |
| isosinensetin | CA1 | naringin | ADORA3 |
| isosinensetin | CA9 | naringin | EIF4A1 |
| isosinensetin | GSK3B | naringin | MAOB |
| isosinensetin | MMP9 | naringin | CYP1B1 |
| isosinensetin | CA6 | naringin | ABCG2 |
| isosinensetin | CA13 | naringin | ABCC1 |
| isosinensetin | PFKFB3 | naringin | HSD17B1 |
| isosinensetin | MMP13 | naringin | SHBG |
| isosinensetin | BACE1 | naringin | CBR1 |
| isosinensetin | PIK3CG | naringin | LGALS1 |
| isosinensetin | CDK2 | naringenin | CYP19A1 |
| isosinensetin | ESR2 | naringenin | CA7 |
| isosinensetin | XDH | naringenin | HSD17B1 |
| isosinensetin | CYP19A1 | naringenin | CA12 |
| isosinensetin | PTK2 | naringenin | SHBG |
| isosinensetin | CCNB3 | naringenin | CA4 |
| isosinensetin | CDK1 | naringenin | CYP1B1 |
| isosinensetin | CCNB1 | naringenin | CBR1 |
| isosinensetin | CCNB2 | naringenin | ESR1 |
| isosinensetin | MMP2 | naringenin | ESR2 |
| isosinensetin | DRD4 | naringenin | PTGS1 |
| isosinensetin | NEK2 | naringenin | MAOB |
| isosinensetin | AXL | naringenin | ADORA3 |
| isosinensetin | CYP1A1 | naringenin | ABCG2 |
| isosinensetin | CD38 | naringenin | TAS2R31 |
| isosinensetin | MAOA | naringenin | AKR1C3 |
| isosinensetin | GLO1 | naringenin | PLA2G1B |
| isosinensetin | ARG1 | naringenin | GRM5 |
| isosinensetin | CAMK2B | naringenin | CES1 |
| isosinensetin | PLG | naringenin | PPARG |
| isosinensetin | ALOX12 | naringenin | CES2 |
| isosinensetin | PLA2G1B | naringenin | MMP12 |
| isosinensetin | APEX1 | naringenin | SLC5A2 |
| isosinensetin | AKR1C1 | naringenin | POLB |
| isosinensetin | AKR1C4 | naringenin | MMP13 |
| isosinensetin | ACHE | naringenin | PLA2G2A |
| isosinensetin | CA14 | naringenin | PLA2G5 |
| isosinensetin | AKT1 | naringenin | PLA2G10 |
| isosinensetin | CA5A | naringenin | BACE1 |
| isosinensetin | PLA2G2A | naringenin | SERPINE1 |
| isosinensetin | MMP12 | naringenin | KLK1 |
| isosinensetin | PTPRS | naringenin | KLK2 |
| isosinensetin | MCL1 | naringenin | RXRA |
| isosinensetin | TNKS | naringenin | CHRNA7 |
| isosinensetin | AVPR2 | naringenin | SRC |
| isosinensetin | IKBKB | naringenin | CA2 |
| isosinensetin | ODC1 | naringenin | CA1 |
| isosinensetin | PPP5C | naringenin | CA3 |
| isosinensetin | HSD17B2 | naringenin | CA6 |
| isosinensetin | TNKS2 | naringenin | CA13 |
| isosinensetin | LRRK2 | naringenin | CA5B |
| isosinensetin | AKR1C3 | naringenin | CA5A |
| isosinensetin | MMP3 | naringenin | KIT |
| isosinensetin | PDPK1 | naringenin | KDR |
| isosinensetin | CNR2 | naringenin | FGFR1 |
| isosinensetin | PIK3CA | naringenin | MET |
| isosinensetin | CYP1A2 | naringenin | NQO2 |
| isosinensetin | SIRT3 | naringenin | HSD17B14 |
| isosinensetin | SIRT2 | naringenin | AKR1B1 |
| isosinensetin | CCND1 | naringenin | BCHE |
| isosinensetin | CDK4 | naringenin | NOX4 |
| isosinensetin | SCD | naringenin | CA9 |
| isosinensetin | HSD11B1 | naringenin | IGF1R |
| isosinensetin | AMY1A | naringenin | INSR |
| narirutin | SLC5A1 | naringenin | CLK1 |
| narirutin | SLC5A4 | naringenin | DYRK1B |
| narirutin | TYR | naringenin | CDK5R1 |
| narirutin | EPHX2 | naringenin | CDK5 |
| narirutin | SRD5A1 | naringenin | ESRRA |
| narirutin | SLC28A3 | naringenin | ESRRB |
| narirutin | MMP1 | naringenin | HSD17B2 |
| narirutin | MMP7 | naringenin | EDNRA |
| narirutin | MMP8 | naringenin | DYRK1A |
| narirutin | EIF4A1 | naringenin | SIRT2 |
| narirutin | ADORA2A | naringenin | IGFBP3 |
| narirutin | ABCC1 | naringenin | PTGER1 |
| geniposide | CA2 | naringenin | PTGER4 |
| geniposide | CA1 | naringenin | PTGER2 |
| geniposide | CA12 | naringenin | PTGER3 |
| geniposide | CA9 | naringenin | PIK3CB |
| geniposide | ADORA1 | naringenin | CYP2C9 |
| geniposide | ADORA2A | naringenin | CYP3A4 |
| geniposide | CA14 | naringenin | PIK3CA |
| geniposide | ADORA3 | naringenin | BCL2L1 |
| geniposide | ADK | naringenin | F3 |
| geniposide | LGALS3 | naringenin | ADCY5 |
| geniposide | LGALS9 | naringenin | PGF |
| geniposide | SLC29A1 | naringenin | VEGFA |
| geniposide | ADA | naringenin | MMP2 |
| geniposide | FUCA1 | naringenin | YWHAG |
| geniposide | GBA | naringenin | CTSL |
| geniposide | SLC5A2 | naringenin | ALOX12 |
| geniposide | ADORA2B | naringenin | MMP3 |
| geniposide | HK2 | naringenin | APP |
| geniposide | HK1 | naringenin | BCL2 |
| geniposide | TYR | naringenin | VCP |
| geniposide | CA7 | naringenin | LCK |
| geniposide | MMP2 | naringenin | SYK |
| geniposide | CA13 | naringenin | WEE1 |
| geniposide | FOLH1 | naringenin | HNF4A |
| geniposide | LGALS4 | geniposide | AKR1C3 |
| geniposide | LGALS8 | geniposide | TYMP |
| geniposide | EIF4H | geniposide | SLC5A4 |
| geniposide | PABPC1 | geniposide | MAG |
| geniposide | SLC5A1 | geniposide | CA4 |
| geniposide | HSPA8 | geniposide | CA5A |
| geniposide | EPHX2 | geniposide | AKR1B1 |

Table S2. GO biological process analysis of ZZHPD

| NO. | GO Term | PValue |
| --- | --- | --- |
| 1 | integral component of plasma membrane | 2.14E-12 |
| 2 | cell junction | 4.88E-09 |
| 3 | postsynaptic membrane | 2.64E-08 |
| 4 | axon | 6.40E-08 |
| 5 | positive regulation of peptidyl-serine phosphorylation | 5.73E-07 |
| 6 | neuron projection | 2.85E-06 |
| 7 | protein serine/threonine kinase activity | 3.73E-06 |
| 8 | response to morphine | 4.87E-06 |
| 9 | prepulse inhibition | 7.76E-06 |
| 10 | ATP binding | 1.48E-05 |
| 11 | negative regulation of adenylate cyclase activity | 1.65E-05 |
| 12 | peptidyl-serine phosphorylation | 2.71E-05 |
| 13 | chemical synaptic transmission | 2.71E-05 |
| 14 | serotonin binding | 3.00E-05 |
| 15 | visual learning | 3.31E-05 |
| 16 | positive regulation of ERK1 and ERK2 cascade | 6.26E-05 |
| 17 | GABA-A receptor complex | 6.82E-05 |
| 18 | protein phosphorylation | 8.38E-05 |
| 19 | intracellular signal transduction | 9.97E-05 |
| 20 | negative regulation of neuron apoptotic process | 1.05E-04 |

Table S3. KEGG pathways enrichment of ZZHPD

| NO. | KEGG Pathway | PValue |
| --- | --- | --- |
| 1 | Serotonergic synapse | 1.07E-14 |
| 2 | Neuroactive ligand-receptor interaction | 2.27E-14 |
| 3 | Rap1 signaling pathway | 1.44E-11 |
| 4 | Glioma | 2.18E-11 |
| 5 | Long-term potentiation | 2.18E-11 |
| 6 | Proteoglycans in cancer | 7.04E-11 |
| 7 | Dopaminergic synapse | 2.25E-10 |
| 8 | Prostate cancer | 5.08E-10 |
| 9 | ErbB signaling pathway | 5.08E-10 |
| 10 | cAMP signaling pathway | 5.26E-10 |
| 11 | Neurotrophin signaling pathway | 2.29E-09 |
| 12 | Central carbon metabolism in cancer | 6.78E-09 |
| 13 | Thyroid hormone signaling pathway | 9.79E-09 |
| 14 | HIF-1 signaling pathway | 2.52E-08 |
| 15 | Non-small cell lung cancer | 5.64E-08 |
| 16 | mTOR signaling pathway | 8.60E-08 |
| 17 | VEGF signaling pathway | 8.60E-08 |
| 18 | Alcoholism | 1.27E-07 |
| 19 | Nicotine addiction | 1.34E-07 |
| 20 | Serotonergic synapse | 1.64E-07 |
